# Geometric Aspects of the Isentropic Liquid Dynamics and Vorticity Invariants

**DOI:** 10.3390/e22111241

**Published:** 2020-10-31

**Authors:** Alexander A. Balinsky, Denis Blackmore, Radosław Kycia, Anatolij K. Prykarpatski

**Affiliations:** 1 School of Mathematics, Cardiff University, Cardiff CF24 4AG, UK; BalinskyA@cardiff.ac.uk; 2 Department of Mathematical Sciences, New Jersey Institute of Technology, Newark, NJ 07102, USA; denblac@gmail.com; 3 Faculty of Physics, Mathematics and Computer Science, Cracow University of Technology, 31-155 Kraków, Poland; kycia.radoslaw@gmail.com; 4 Department of Physics, Mathematics and Computer Science, Cracov University of Technology, 31-155 Kraków, Poland

**Keywords:** liquid flow, hydrodynamic Euler equations, diffeomorphism group, Lie-Poisson structure, isentropic hydrodynamic invariants, vortex invariants, charged liquid fluid dynamics, symmetry reduction, 11.10.Ef, 11.15.Kc, 11.10.-z, 11.15.-q, 11.10.Wx, 05.30.-d

## Abstract

We review a modern differential geometric description of fluid isentropic motion and features of it including diffeomorphism group structure, modelling the related dynamics, as well as its compatibility with the quasi-stationary thermodynamical constraints. We analyze the adiabatic liquid dynamics, within which, following the general approach, the nature of the related Poissonian structure on the fluid motion phase space as a semidirect Banach groups product, and a natural reduction of the canonical symplectic structure on its cotangent space to the classical Lie-Poisson bracket on the adjoint space to the corresponding semidirect Lie algebras product are explained in detail. We also present a modification of the Hamiltonian analysis in case of a flow governed by isothermal liquid dynamics. We study the differential-geometric structure of isentropic magneto-hydrodynamic superfluid phase space and its related motion within the Hamiltonian analysis and related invariant theory. In particular, we construct an infinite hierarchy of different kinds of integral magneto-hydrodynamic invariants, generalizing those previously constructed in the literature, and analyzing their differential-geometric origins. A charged liquid dynamics on the phase space invariant with respect to an abelian gauge group transformation is also investigated, and some generalizations of the canonical Lie-Poisson type bracket is presented.

## 1. Introduction

Our review is devoted to compressible liquid or gas motions in which entropy remains locally constant throughout the flowfield, i.e., the flow for which the entropy of a moving element along a streamline remains constant, is called isentropic. This means that along different streamlines, the entropy changes normal to the streamlines. As a typical example, one can mention the flowfield behind a curved shock wave, where streamlines, passing through different locations along the curved shock wave, experience different increases in entropy. Hence, downstream from this shock, the entropy can be constant along a given streamline but differs from one streamline to another. Namely this type of flow, with entropy constant along streamlines, is defined as isentropic. Flow with entropy constant everywhere is then called homentropic. Here we need to remark that owing to the second law of thermodynamics, an isentropic flow does not strictly exist. We know from thermodynamics well that an isentropic flow is defined to be along streamlines both adiabatic and reversible. Yet, all real flows always experience to some extent the irreversible phenomena of friction, thermal conduction, and diffusion. For instance, any nonequilibrium, chemically reacting flow is always irreversible, when considered to be a closed system. Nonetheless, there are a large number of liquid and gas dynamic problems with entropy increase negligibly slight, which for the purpose of analysis are assumed to be isentropic. Examples are flows through subsonic and supersonic nozzles, as in wind tunnels and rocket engines, or shock-free flows over a wing, fuselage, or other aerodynamic shapes. For all of them, except for a flow near the thin boundary-layer region, adjacent to the surface where friction and thermal conduction effects can be strong, the outer inviscid flow can be considered isentropic. In contrast, if shock waves exist in the flow, the entropy increase across these shocks destroys the assumption of isentropic flow, although the flow along streamlines between shocks may persist to be isentropic.

As an isentropic flow is governed by thermodynamically reversible processes, being adiabatic along a streamline, it needs to be specified with locally defined thermodynamical parameters, such as the medium density ρ, the specific entropy σ, the local medium absolute temperature T, the pressure *p* and the specific energy e. All these quantities are related to each other some way, which can be retrieved following the classical Gibbs reasonings. Namely we assume from the very beginning that the reversible thermodynamical state of the medium under regard is completely locally described by means of the following first pair: (p-local *pressure*,ρ*-specific density*) of thermodynamical parameters. Assume now that the same thermodynamical state of this medium can be also simultaneously described by means of the following second pair: (T-local absolute temperature, σ-specific entropy). The latter, in particular, means that a suitable functional transformation from one pair of parameter to another, if smooth, is diffeomorphic, which is the Jacobian $J_{\left(\sigma ,T\right)}\left(p,\rho \right)$ of this transformation $R_{+}^{2}∋\left(\sigma ,T\right)\to \left(p,\rho \right)\in R_{+}^{2}$ is not degenerate everywhere, i.e.,

(1) $$J_{\left(\sigma ,T\right)}\left(p,\rho \right)=\frac{\partial (p,\rho )}{\partial (\sigma ,T)}:=\det\left(\begin{array}{cc} \frac{\partial p}{\partial \sigma } & \frac{\partial p}{\partial T}\\ \frac{\partial \rho }{\partial \sigma } & \frac{\partial \rho }{\partial \sigma } \end{array}\right)\neq 0\;$$

at all points $\left(\sigma ,T\right)\in R_{+}^{2}.$ Taking into account that the local absolute temperature *T* and the adiabatic σ parameters are, in general, defined with some scaling ambiguity, we can always put, by definition that $J_{\left(\sigma ,T\right)}\left(p,\rho \right)=\rho^{2}\neq 0$ everywhere. As a simple consequence of multiplying this expression by the unity Jacobian $J_{\left(\sigma ,\rho \right)}\left(\sigma ,\rho \right)=1$ one easily derives that

(2) $$\begin{array}{c} J_{\left(\sigma ,T\right)}\left(p,\rho \right)\times J_{\left(\sigma ,\rho \right)}\left(\sigma ,\rho \right)=\;\\ =\frac{\partial (p,\rho )}{\partial (\sigma ,T)}\frac{\partial (\sigma ,\rho )}{\partial (\sigma ,\rho )}=\frac{\partial (\rho ,p)}{\partial (\sigma ,\rho )}\frac{\partial (\sigma ,\rho )}{\partial (\sigma ,T)}=\rho^{2}, \end{array}$$

or, equivalently,

(3) $$\frac{\partial (p,\rho )}{\partial (\sigma ,\rho )}=\rho^{2}\frac{\partial (\sigma ,T)}{\partial (\sigma ,\rho )}⟺{\left.\frac{\partial (p/\rho^{2})}{\partial \sigma }\right|}_{\rho }={\left.\frac{\partial T}{\partial \rho }\right|}_{\sigma }$$

at all points $\left(\sigma ,\rho \right)\in R_{+}^{2}.$ The equality of partial derivatives above simply means, owing to the well known Montel-Menchoff-Young theorem [1,2,3], the existence of such a differentiable thermodynamic state function $R_{+}^{2}∋\left(\rho ,\sigma \right)\to e\in R,$ that its differential satisfies the following equality:

(4) $$\delta e\left(\rho ,\sigma \right)=T\delta \sigma +p\delta \rho /\rho^{2}.$$

The latter expression presents exactly the written down second thermodynamic law with respect to the locally defined variables, if the smooth function $R_{+}^{2}∋\left(\rho ,\sigma \right)\to e\in R$ is interpreted as the specific medium energy of the system at the internal absolute temperature T=T(ρ,σ) and pressure p(ρ,σ) at suitably fixed state parameters $\left(\rho ,\sigma \right)\in R_{+}^{2}.$ Taking in addition that our medium is imbedded into some domain $M\subset R^{3},$ moving in space-time, our next task is to describe adequately the related motion spatial phase space variables, compatible with the corresponding Euler evolution equations.

## 2. Spatial Phase Space Description

It is well known that the same physical system is often described using different sets of variables, related with their different physical interpretation. Simultaneously, this same system is endowed with different mathematical structures deeply depending on the geometric scenario used for its description. In general, these structures prove to be not equivalent but some special way connected to each other. In particular, such double descriptions commonly occur in systems with distributed parameters as hydrodynamics, magnetohydrodynamics and diverse gauge systems, which are effectively described by means of both symplectic and Poissonian structures on suitable phase spaces. In particular, it was observed [4,5,6,7,8,9,10,11] that these structures are canonically related to each other. Mathematical properties, lying in a background of their analytical description, make it possible to study additional important parameters [12,13,14,15,16,17,18,19,20,21,22,23,24,25,26] of different hydrodynamic and magnetohydrodynamic systems, amongst which we will mention integral invariants, describing such internal fluid motion peculiarities as vortices, topological singularities and other different instability states, strongly depending [27,28] on imposed isentropic fluid motion constraints. Being interested in their general properties and mathematical structures, responsible for their existence and behavior, we present a detail enough differential geometrical approach to investigating thermodynamically quasi-stationary isentropic fluid motions, paying more attention to analytical argumentation of tricks and techniques used during the presentation.

In particular, we consider a compressible liquid filling a compact linearly-connected domain M⊂$R^{3}$ with smooth boundary ∂M, and moving free of external forces. A configuration of this fluid is called the reference or Lagrangian configuration, its points are called material or Lagrangian points and denoted by X∈M and are referred as material, or Lagrangian coordinates. We shall not for now be specific about the correct choices of the related functional spaces to be used and refer to works [29,30], where this is discussed in great detail. The manifold $M\subset R^{3},$ thought of as the target space of a configuration η∈Diff(M) of the fluid at a different time, is called the spatial or Eulerian configuration, whose points, called spatial or Eulerian points, will be denoted by small letters x∈M. Then a motion of the fluid is a time dependent family [4,7,19,26,30,31,32,33,34] of diffeomorphisms written as

(5) $$M∋x_{t}\;=\eta \left(X,t\right):=\eta_{t}\left(X\right)\in M$$

for any initial configuration X∈M and some mapping $\eta_{t}\in Diff\left(M\right),t\in R.$ We also are given the mass density $\rho_{0}\;\in R\left(M\right)\subset C^{\infty }\left(M;R_{+}\right)\;$ and the specific entropy $\sigma_{0}\in \Sigma (M)\subset$$C^{\infty }\left(M;R_{+}\right)$ of the fluid in the reference configuration, changing in time in such a way that

(6) $$\rho_{0}\left(X\right)=\rho_{t}\left(x_{t}\right)J_{\eta_{t}}\left(x_{t}\right),\sigma_{0}\left(X\right)=\sigma_{t}\left(x_{t}\right),$$

where $J_{\eta_{t}}\left(x_{t}\right)$ denotes the standard Jacobian determinant of the motion $\eta_{t}\in Diff\left(M\right)$ at $x_{t}\in M$ and $\sigma_{t}\left(x_{t}\right)\;$ denotes the specific entropy for any $x_{t}=\eta_{t}\left(X\right)\in M$ and t∈R. For a motion $x_{t}=\eta_{t}\left(X\right)\in M$ and arbitrary X∈M,t∈R, one usually defines three velocities:

the material or Lagrangian velocity

(7) $$V\left(X,t\right)=V_{t}\left(X\right):=\partial \eta_{t}\left(X\right)/\partial t,$$

the spatial or Eulerian velocity

(8) $$v\left(x_{t},t\right)=v_{t}\left(x_{t}\right):=v_{t}\circ \eta_{t}\left(X\right)$$

and convective or body velocity

(9) $$V\left(X,t\right)=V_{t}\left(X\right):=-\partial X\left(x_{t},t\right)/\partial t=-\partial \eta_{t}^{-1}\left(x_{t}\right)/\partial t,\;$$

being equivalent to the expression $V_{t}=\;\eta_{t,*}^{-1}v_{t}$ for all t∈R. Since the velocity $v_{t}:M\in T\left(M\right)$ is tangent to *M* for all t∈R at $\;x_{t}=\eta_{t}\left(X\right)\in M,$ it determines a time dependent vector field on M. On the other hand, tangency of $V_{t}\left(X\right)$ and $\eta_{t}\left(X\right),X\in M,$ means that the velocity $V_{t}$ is a vector field over a configuration $\eta_{t}\in Diff\left(M\right)$ on M, that is $V_{t}:M\to T\left(M\right)$ is such a map that $V_{t}\left(X\right)$ is tangent to *M* not at X∈M, but at point $x_{t}=\eta_{t}\left(X\right)\in M.$ Simultaneously the velocity $V_{t}\left(X\right)$ is a tangent vector to *M* at X∈M, that is $V_{t}$ is also a time dependent vector field on M. In what will follow we will think of the fluid as moving smoothly in the domain $M\subset R^{3},$ at any time filling it and producing no shocks and cavitation.

We present in Section 3 an introductory section with a modern differential geometric description of the isentropic fluid motion phase space and featuring diffeomorphism group structure, modelling the related dynamics, as well as its compatibility with the quasi-stationary thermodynamical constraints. Section 4 is devoted to the Hamiltonian analysis of the adiabatic liquid dynamics, within which, following the general approach of [6,19,33], we explain the nature of the related Poissonian structure on the fluid motion phase space, as a semidirect Banach groups product, and a natural reduction of the canonical symplectic structure on its cotangent space to the classical Lie-Poisson bracket on the adjoint space to the corresponding semidirect Lie algebras product. A modification of the Hamiltonian analysis in case of the isothermal liquid dynamics is presented in Section 5. In Section 6 we proceed to studying the differential-geometric structure of the adiabatic magneto-hydrodynamic superfluid phase space and its related motion within the Hamiltonian analysis and invariant theory. We construct there an infinite hierarchy of different kinds of integral magneto-hydrodynamic invariants, generalizing those previously constructed in [33,35], and analyzing their differential-geometric origins. The last section, Section 7, presents a charged fluid dynamics on the phase space invariant with respect to an abelian gauge group transformation.

## 3. Ideal Liquid Dynamics and Its Geometry

It is well known that the motion of an ideal compressible and isentropic fluid is governed by the Euler equations

(10) $$\begin{array}{c} \partial v/\partial t+\langle v|\nabla \rangle v+\rho^{-1}\nabla p^{\left(0\right)}=0,\\ \partial \rho /\partial t+\langle \nabla |\rho v\rangle =0,\partial \sigma /\partial t+\langle v|\nabla \rangle \sigma =0, \end{array}$$

where $p_{0}:M\to R$ is the internal fluid pressure, $\sigma =\sigma \left(x_{t},t\right)=\sigma_{t}\left(x_{t}\right)$ is the specific entropy at a spatial point $x_{t}=\eta_{t}\left(X\right)\in M$ for any t∈R, which is fixed owing to the Euler Equation (10), ∇:=∂/∂x is the usual gradient on the space of smooth functions $C^{\infty }\left(M;R\right)$ and 〈·|·〉 denotes the usual convolution on T(M)×T(M) subject to the usual metric in $R^{3},$ reduced on the submanifold M. The evolution (10) is considered to be *a priori* thermodynamically quasi-stationary, which is the following *infinitesimal heat convective* and strictly mathematical relationship (4), derived above in Introduction,

(11) $$\;\delta e_{t}\left(\rho_{t}\left(x_{t}\right),\sigma_{t}\left(x_{t}\right)\right)=T_{t}\left(x_{t}\right)\delta \sigma_{t}\left(x_{t}\right)+p_{t}^{\left(0\right)}\left(x_{t}\right)\rho_{t}^{-2}\left(x_{t}\right)\delta \rho_{t}\left(x_{t}\right)\;$$

holds for all $x_{t}\in M$ and t∈R, where $\;\;e_{t}:R\left(M\right)\times \Sigma \left(M\right)\to C^{\infty }\left(M\times R;R\right)$ denotes the internal specific fluid energy, $T_{t}:\;M\;\to \;R_{+}\;$ denotes the internal fluid absolute temperature, $p_{t}^{\left(0\right)}:M\to R$ is the internal liquid pressure and the variation sign “δ” means the change subject to both the temporal variable t∈R and the spatial variable $x_{t}\in M.$

Let us now analyze the internal mathematical structure of quantities $\left(\rho_{t},\sigma_{t}\right)\in R\left(M\right)\times \Sigma \left(M\right)$ as the *physical observables* subject to their evolution (10) with respect to the group diffeomorphisms $\eta_{t}\in \;Diff\left(M\right),t\in \;R,$ generated by the liquid motion vector field $dx_{t}/dt=v_{t}\left(x_{t}\right),x_{t}:=\eta_{t}\left(X\right),t\in R,$X∈M:

(12) $$\begin{array}{c} L_{d/dt}\left(\rho_{t}d^{3}x_{t}\langle v_{t}|dx_{t}\rangle \right)=\rho_{t}d^{3}x_{t}(-\rho_{t}^{-1}dp_{t}^{\left(0\right)}+d|v_{t}{|}^{2}/2),\;\\ L_{d/dt}\left(\rho_{t}d^{3}x_{t}\right)=0,\;\;L_{d/dt}\sigma_{t}=0, \end{array}$$

where $L_{d/dt}:\Lambda \left(M\right)\to \Lambda \left(M\right)$ denotes the corresponding Lie derivative with respect to the vector field $d/dt:=\partial /\partial t+\langle v_{t}|\nabla \rangle \in \Gamma \left(M\times R;T\left(M\right)\right),t\in R.$ The relationships (12) here simply mean that at every fixed t∈R the space of physical observables, being by definition, the adjoint space $G^{*}:=\;\left(\Lambda^{1}\left(M\right)⊗\Lambda^{3}\left(M\right)\right)⊕(\Lambda^{3}\left(M\right)$$⊕\Lambda^{0}\left(M\right))\;$ to the vector space $G:=\Gamma \left(M;T\left(M\right)\right)\times \left(\Lambda^{0}\left(M\right)⊕\Lambda^{3}\left(M\right)\right)$$≃T_{Id}\left(G\right),$ the tangent space at the identity Id to the extended differential-functional group manifold $\;G:=Diff\left(M\right)\times (\Lambda^{0}\left(M\right)$$\times \Lambda^{3}\left(M\right))$≃Diff(M)×(R(M)×Σ(M)), where we have naturally identified the abelian group product $\Lambda^{0}\left(M\right)$$\times \Lambda^{3}\left(M\right)$ with its direct tangent space sum $T(\Lambda^{0}\left(M\right))$$⊕T(\Lambda^{3}\left(M\right)).$

Consider now the natural action Diff(M)×G→G of the Diff(M)-group on the constructed differential-functional manifold G:

(13) $$\begin{array}{c} (\eta \circ \varphi )(X):=\varphi (\eta (X)),(\eta \circ r)(X):=\;r(\eta (X)),\;\\ \eta \circ \;\left(s\left(X\right)d^{3}X\;\right):=\;\eta^{*}\left(s\left(X\right)d^{3}X\right) \end{array}$$

for η∈Diff(M),X∈M and any (φ;r,s)∈Diff(M)×(R(M)×Σ(M)). Then, taking into account the suitably extended action (13) on the differential-functional manifold G, one can formulate the following easily checkable and crucial for what will follow further proposition.

**Proposition** **1.** *The functional manifold G:=Diff(M)×(R(M)×Σ(M)) in Eulerian coordinates is a smooth symmetry Banach group G:=Diff(M)⋉(R(M)×Σ(M)), equal to the semidirect product of the diffeomorphism group Diff(M) and the direct product R(M)×Σ(M) of abelian functional $R\left(M\right)≃\;\Lambda {\;}^{0}\left(M\right),$ and density $\;\;\Sigma \left(M\right)≃\Lambda^{3}\left(M\right)$ group, endowed in Eulerian variables with the following right group multiplication law:*

(14) $$\begin{array}{c} \left(\varphi_{1};r_{1},s_{1}d^{3}x\right)\circ \left(\varphi_{2};r_{2},s_{2}d^{3}x\right)=\;\\ =(\varphi_{2}\cdot \varphi_{1};r_{1}+r_{2}\cdot \varphi_{1},s_{1}d^{3}x+\left(s_{2}d^{3}x\right)\cdot \varphi_{1}) \end{array}$$

*for arbitrary elements $\varphi_{1},\varphi_{2}\in Diff\left(M\right),r_{1},r_{2}\in \Lambda^{0}\left(M\right)$ and $\;s_{1}d^{3}x,s_{2}d^{3}x$$\in \Lambda^{3}\left(M\right).$*

This proposition allows a simple enough interpretation, namely, it means that the adiabatic mixing of the $G∋(\varphi_{2};r_{2},s_{2}d^{3}x)$-liquid configuration with the $G∋(\varphi_{1};r_{1},s_{1}d^{3}x)$-liquid configuration amounts to summation of their densities and entropies, simultaneously changing the common specific density owing to the fact that some space of the domain *M* is already occupied by the first liquid configuration and the second one should be diffeomorphically shifted from this configuration to another free part of the spatial domain M, whose volume is assumed to be fixed and bounded.

The second important observation concerns the variational one-form (11) which can be naturally interpreted as some constraint on the group manifold *G* for any fixed initial extended Lagrangian configuration $(\eta ;\rho_{0},\sigma_{0}d^{3}X)\in G,$ as it follows from the conditions (6):

(15) $$\;J_{\eta_{t}}\left(X\right)\rho_{t}\circ \eta_{t}\left(X\right):=\rho_{0}\left(X\right),\;\sigma_{t}\circ \eta_{t}\left(X\right):=\sigma_{0}\left(X\right)\;$$

for all X∈M,$\eta_{t}\in Diff\left(M\right)$ and t∈R. In addition, if to determine, owing to (11) and the streamline adiabatic constraint $\delta \sigma_{t}\left(x_{t}\right)=0\;$ for all t∈R, the specific energy density

(16) $$e_{t}\left(\rho_{t},\sigma_{t}\right):=w_{t}^{\left(0\right)}\left(\rho_{t},\sigma_{t}\right)+c_{t}\left(\sigma_{t}\right)\;$$

for some still unknown mapping $c_{t}:\;\Sigma \left(M\right)\to C^{\infty }\left(M\times R;R\right)$ and the internal potential energy function $w_{t}^{\left(0\right)}:R\left(M\right)\times \Sigma \left(M\right)\to C^{\infty }\left(M;R\right)$ of the liquid under regard, the local energy conservation property

(17) $$\;\frac{d}{dt}\int_{D_{t}}e_{t}\left(\rho_{t},\sigma_{t}\right)\rho_{t}\left(x_{t}\right)d^{3}x_{t}=-\int_{D_{t}}\langle \nabla |p_{t}^{\left(0\right)}\left(x_{t}\right)v_{t}\left(x_{t}\right)\rangle d^{3}x_{t}$$

holds for all t∈R and the domain $D_{t}:=\eta_{t}(D$)⊂M, where a smooth submanifold *D*⊂M is chosen arbitrary and $\eta_{t}:$M→M denotes the corresponding evolution subgroup of the diffeomorphism group $Diff_{0}\left(M\right),$ generated by the Euler evolution Equation (10), becomes compatible with constraint (11) iff there holds the following equality:

(18) $$p_{t}^{\left(0\right)}\left(x_{t}\right)=\rho_{t}{\left(x_{t}\right)}^{2}\partial w_{t}^{\left(0\right)}\left(\rho_{t},\sigma_{t}\right)/\partial \rho_{t}\;$$

for all $x_{t}\in M$ and t∈R. In particular, from (17) and (18) the following global internal energy functional

(19) $$H:=\int_{M}\left[w_{t}^{\left(0\right)}\left(\rho_{t},\sigma_{t}\right)+c_{t}\left(\sigma_{t}\right)\right]\;\rho_{t}\left(x_{t}\right)d^{3}x_{t}$$

is conserved that is dH/dt=0 for all t∈R.

As the extended Lagrangian configuration $(\eta ;\rho_{0},\sigma_{0}d^{3}X)\in G$ is fixed for all whiles of time t∈R and the dynamical variables $\rho_{t}\in R\left(M\right)\;$ and $\sigma_{t}\in \Sigma \left(M\right)$ depend only on the evolution diffeomorphisms $\eta_{t}\in Diff\left(M\right),$t∈R, it is reasonable to consider the constraint (11) as an element of the cotangent space $T_{\eta_{t}}^{*}\left(Diff\left(M\right)\right)$ to the diffeomorphism group Diff(M) at the point $\eta_{t}\in Diff\left(M\right)$ for any t∈R.

Determine first the tangent space $T_{\eta }\left(G\right)$ to the group manifold *G* at point $(\eta ;\rho_{0},\sigma_{0}d^{3}X)\in G,$ which will be the direct product of the tangent spaces $T_{\eta }\left(Diff\left(M\right)\right),T_{\rho_{0}}\left(\Lambda^{0}\left(M\right)\right)$ and $T_{\sigma_{0}d^{3}X}\left(\Lambda^{3}\left(M\right)\right).$ The last two tangent spaces are isomorphic, respectively, to themselves that is $T_{\rho_{0}}\left(\Lambda^{0}\left(M\right)\right)≃\;\;\Lambda^{0}\left(M\right)$ and $\;T_{\sigma_{0}d^{3}X}\left(\Lambda^{3}\left(M\right)\right)≃\;\Lambda^{3}\left(M\right)$ at any X∈M. Their adjoint spaces are naturally determined as suitably constructed density and functional spaces on the manifold $M:T_{\rho_{0}}^{*}\left(\Lambda^{0}\left(M\right)\right)≃$$\Lambda^{3}\left(M\right)\;$ and $T_{\sigma_{0}d^{3}X}^{*}\left(\Lambda^{3}\left(M\right)\right)≃\;\Lambda^{0}\left(M\right).$ Concerning the tangent space $T_{\eta }\left(Diff\left(M\right)\right)$ at a configuration η∈Diff(M) we will make use of the construction, devised before in [31,33,36]. Namely, let η∈Diff(M) be a Lagrangian configuration and determine the tangent space $T_{\eta }\left(Diff\left(M\right)\right)$ at η∈Diff(M) as the collection of left invariant vectors $\xi_{\eta }:=L_{\eta ,*}\xi$ at η∈Diff(M), where $L_{\eta }:Diff\left(M\right)\to Dif\left(M\right)$ is, by definition, the left shift on the diffeomorphism group Diff(M) and $\xi \in T_{Id}\left(Diff\left(M\right)\right)$ is a tangent vector at the unity Id∈Diff(M). It is obvious that for all reference points X∈M and any smooth curve R∋τ→$\eta_{\tau }\in Diff\left(M\right)$ of diffeomorphisms of *M* the set of right invariant vectors $\xi \left(X\right)=\;\left(\eta^{-1}\circ d\eta_{t}/d\tau \right)\left(X\right){)|}_{\tau =0}$$\in T_{X}\left(M\right)\;\;$ at point X∈M defines a smooth vector field ξ:M→T(M) on the manifold M. Since, by definition, the tangent space $T_{Id}\left(Diff\left(M\right)\right)$ coincides with the Lie algebra diff(M) of the diffeomorphism group Diff(M), strictly isomorphic to the Lie algebra Γ(T(M)) of right invariant vector fields on M, the dual space $T_{Id}^{*}\left(Diff\left(M\right)\right)$ can be naturally determined from the geometric point of view as the space $diff^{*}\left(M\right),$ consisting of analytic functions on diff(M) and coinciding with the set of one-form densities on M:

(20) $$diff^{*}\left(M\right)≃\Lambda^{1}\left(M\right)⊗\left|\Lambda^{3}\left(M\right)\right|.$$

Similarly, the cotangent space $T_{\eta }^{*}\left(Diff\left(M\right)\right)$ consists of all one-form densities on *M* over η∈Diff(M):

(21) $$T_{\eta }^{*}\left(Diff\left(M\right)\right)=\{\alpha_{\eta }:M\to T^{*}\left(M\right)⊗|\Lambda^{3}\left(M\right)|:\alpha_{\eta }\left(X\right)\in T_{\eta (X)}^{*}\left(M\right)⊗|\Lambda^{3}\left(M\right)\left|\right\}$$

subject to the canonical nondegenerate convolution ${\left(\cdot |\cdot \right)}_{c}$ on $T_{\eta }^{*}\left(Diff\left(M\right)\right)\times T_{\eta }\left(Diff\left(M\right)\right):$ if $\alpha {\;}_{\eta }\in \;$$T_{\eta }^{*}\left(Diff\left(M\right)\right),$$\xi_{\eta }\in T_{\eta }\left(Diff\left(M\right)\right),$ where $\alpha_{\eta }{|}_{X}=\;\langle \alpha_{\eta }\left(X\right)|dx\rangle ⊗d^{3}X,$$\xi_{\eta }{|}_{X}=\;\langle \xi_{\eta }\left(X\right)|\partial /\partial x\rangle ,$ then

(22) $${\left(\alpha_{\eta }|\xi_{\eta }\right)}_{c}:=\int_{M}\langle \alpha_{\eta }\left(X\right)|\xi_{\eta }\left(X\right)\rangle d^{3}X.$$

The construction above makes it possible to identify the cotangent bundle $T_{\eta }^{*}\left(Diff\left(M\right)\right)$ at the fixed Lagrangian configuration η∈Diff(M) to the tangent space $T_{\eta }\left(Diff\left(M\right)\right),$ insomuch as the tangent space T(M) is endowed with the natural internal tangent bundle metric 〈·|${\cdot \rangle }_{g}$ at any point η(X)∈M, identifying T(M) with $T^{*}\left(M\right)$ via the metric isomorphism $♯:T^{*}\left(M\right)\to T\left(M\right).$ The latter can be also naturally lifted to $T_{\eta }^{*}\left(Diff\left(M\right)\right)$ at η∈Diff(M), namely: for any elements $\alpha_{\eta },\beta_{\eta }\in T_{\eta }^{*}\left(Diff\left(M\right)\right),\alpha_{\eta }{|}_{X}=\;\langle \alpha_{\eta }\left(X\right)|dx\rangle ⊗d^{3}X$ and $\beta_{\eta }{|}_{X}\;=\langle \beta_{\eta }\left(X\right)|dx\rangle ⊗d^{3}X$$\in T_{\eta }^{*}\left(Diff\left(M\right)\right)$ we can define the metric

(23) $${\left(\alpha_{\eta }|\beta_{\eta }\right)}_{g}:=\int_{M}\rho_{0}\left(X\right){\langle \alpha_{\eta }^{♯}\left(X\right)|\beta_{\eta }^{♯}\left(X\right)\rangle }_{g}d^{3}X,$$

where, by definition, $\;\;\alpha_{\eta }^{♯}\left(X\right):=♯\left(\rho_{0}{\left(X\right)}^{-1}\langle \alpha_{\eta }\left(X\right)|dx\rangle \right),$$\beta_{\eta }^{♯}\left(X\right):=$$♯(\rho_{0}{\left(X\right)}^{-1}\langle \beta_{\eta }\left(X\right)|dx\rangle )$$\in T_{\eta (X)}\left(M\right)$ for any X∈M.

The diffeomorphism group Diff(M) can be naturally restricted to the factor-group $Diff_{0}\left(M\right):=Diff\left(M\right)/Diff_{\rho_{0},\sigma_{0}}\left(M\right)$ subject to the stationary normal symmetry subgroup $\;Diff_{\rho_{0},\sigma_{0}}\left(M\right)\subset Diff\left(M\right),$ where

(24) $$Diff_{\rho_{0},\sigma_{0}}\left(M\right):=\left\{\varphi \in Diff\left(M\right):\rho_{0}\left(X\right)=J_{\varphi (X)}\rho_{0}\left(\varphi \left(X\right)\right),\sigma_{0}\left(X\right)=\sigma_{0}\left(\varphi \left(X\right)\right)\right\}$$

for any X∈M. Based on the construction above one can proceed to constructing smooth flows and functionals on the specially extended group manifold $G_{0}:=Diff_{0}\left(M\right)⋉$$(\Lambda^{0}\left(M\right)\times$$\Lambda^{3}\left(M\right))$ and consider their coadjoint action on the cotangent bundle $T_{g_{\eta }}^{*}\left(G_{0}\right),g_{\eta }:=\left(\eta ;\rho_{0},\sigma_{0}\right)\in G_{0},$ and relate them some way to the evolution with respect to the Euler Equation (10). Moreover, as the cotangent bundle $T_{g_{\eta }}^{*}\left(G_{0}\right),g_{\eta }\in G_{0},$ is a priori endowed with the canonical Poisson structure, one can study both the Hamiltonian flows on it, related with the Euler Equation (10), and a hidden geometrical meaning of the differential constraints like (11).

## 4. Hamiltonian Analysis: The Adiabatic Liquid Dynamics

We observed above that the liquid motion is adequately described by means of the symmetry diffeomorphism group $Diff_{0}\left(M\right),$ acting on the target manifold $M\subset R^{3},$ and this way modeling liquid motion, generated by suitable vector fields on $Diff_{0}\left(M\right).$ This also means that the fluid motion strongly depends on the constraint (11) on the cotangent bundle $T_{g_{\eta }}^{*}\left(G_{0}\right),$$g_{\eta }\in G_{0},$ and *a priori* possesses the canonical Poisson structure on it. Taking into account that the diffeomorphism group $Diff_{0}\left(M\right)$ acts on the extended group density manifold $G_{0}:=Diff_{0}\left(M\right)⋉\left(\Lambda^{0}\left(M\right)\times \Lambda^{3}\left(M\right)\right),$ fixed by the element $(\eta ;\rho_{0},\sigma_{0}d^{3}X)\in G,$ one can suitably construct the canonical Poisson bracket on the cotangent bundle $T_{g_{\eta }}^{*}\left(G_{0}\right),g_{\eta }\in G_{0},$ using the canonical coordinate variables on it. Namely, let $\left(\mu_{\eta };\rho_{0}d^{3}X,\sigma_{0}\right)$$\in T_{g_{\eta }}^{*}\left(G_{0}\right),$$g_{\eta }\in G_{0},$ be coordinates on $T_{g_{\eta }}^{*}\left(G_{0}\right),$ where

(25) $$\begin{array}{ccc} \mu_{\eta }\left(X\right) & = & \rho_{0}\left(X\right)\left[V_{\eta }^{♭}\left(X\right)\right]d^{3}{X|}_{x=\eta (X)}=\\ & = & \rho_{0}\left(X\right)v^{♭}\left(\eta \left(X\right)\right)J_{\eta^{-1}}\left(x\right)d^{3}x:=\rho \left(x\right)v\left(x\right)d^{3}x,\\ r_{\eta }\left(X\right) & = & \rho_{0}\left(X\right)d^{3}X=\rho_{0}\left(X\right)d^{3}{X|}_{x=\eta (X)}:=\rho \left(x\right)d^{3}x,\\ s_{\eta }\left(X\right) & = & \sigma_{0}{\left(X\right)=\sigma \left(\eta \left(X\right)\right)|}_{x=\eta (X)}:=\sigma \left(x\right)\;\; \end{array}$$

and $♭:={♯}^{-1},$ being suitably represented into the Eulerian spatial variables on $T_{g_{\eta }}^{*}\left(G_{0}\right)\;$ at point $\left(\eta ;\rho ,\sigma d^{3}x\right)\in G_{0}.$ In particular, the quantities $\mu \left(x\right):=\rho \left(x\right)v\left(x\right)d^{3}x=\left(\eta^{*}\mu_{\eta }\right)\left(X\right),$$r\left(x\right):=\rho \left(x\right)d^{3}x=\left(\eta^{*}r_{\eta }\right)\left(X\right)$ and $s\left(x\right):=\sigma \left(x\right)=\left(\eta^{*}s_{\eta }\right)\left(X\right)$ are called, respectively, the Eulerian momentum density, the Eulerian fluid density and entropy variables at point x=η(X)∈M. The corresponding metric on $T_{g_{\eta }}^{*}\left(G_{0}\right)$ is given by the expression

(26) $$\begin{array}{c} \left(\left(\alpha_{\eta ,1};r_{\eta ,1}s_{\eta ,1}\right)|\left(\alpha_{\eta ,2};r_{\eta ,2}s_{\eta ,2}\right)\right):=\left(\alpha_{\eta ,1}|\alpha_{\eta ,2}\right)+\;\\ +\left(r_{\eta ,1}|r_{\eta ,2}\right)+\left(s_{\eta ,1}|s_{\eta ,2}\right), \end{array},$$

where $\left(\alpha_{\eta ,1}|\alpha_{\eta ,2}\right)$ for $\alpha_{\eta ,1},\alpha_{\eta ,2}\in T_{\eta }^{*}\left(Diff_{0}\left(M\right)\right)$ is determined by (23) and for any $r_{\eta ,1},r_{\eta ,2}\in T_{\eta }^{*}\left(\Lambda^{3}\left(M\right)\right)$ and $s_{\eta ,1},s_{\eta ,2}\in T_{\eta }^{*}\left(\Lambda^{0}\left(M\right)\right)$ one determines, respectively, as

(27) $$\left(r_{\eta ,1}|r_{\eta ,2}\right):=\int_{M}\;\left(\rho_{1}\left(x\right)\rho_{2}\left(x\right)\right)d^{3}x,\;\left(s_{\eta ,1}|s_{\eta ,2}\right):=\int_{M}\left(\sigma_{1}\left(x\right)\sigma_{2}\left(x\right)\right)d^{3}x.$$

Consider now the cotangent bundle $T_{g_{\eta }}^{*}\left(G_{0}\right)$ at point $\;g_{\eta }=$$\left(\eta ;\rho ,\sigma d^{3}x\right)\in G_{0}$ as a smooth manifold endowed with the canonical symplectic structure on it, equivalent to the corresponding canonical Poisson bracket on $T_{g_{\eta }}^{*}\left(G_{0}\right).$ Taking into account that the manifold $T_{g_{\eta }}^{*}\left(G_{0}\right),$ shifted by the right $R_{\eta^{-1}}$-action to the manifold $T_{Id}^{*}\left(G_{0}\right),$$Id\in G_{0},$ becomes diffeomorphic to the adjoint space $G^{*}$ to the Lie algebra G of the group $G_{0},$ as there was stated [8,9,10,11,19] still by S. Lie in 1887, this canonical Poisson bracket on $T_{\eta }^{*}\left(G_{0}\right)$ transforms [4,10,11,19,34,36] into the classical Lie-Poisson bracket on the adjoint space $G^{*}.$ Moreover, the orbits of the group $G_{0}$ on $T_{g_{\eta }}^{*}\left(G_{0}\right),g_{\eta }=$ $\left(\eta ;\rho ,\sigma d^{3}x\right)\in G_{0},$ transform into the corresponding coadjoint orbits on the adjoint space $G^{*},$ generated by elements of the Lie algebra G. To construct this Lie-Poisson bracket, we formulate preliminary the following proposition.

**Proposition** **2.** *The Lie algebra $G≃\;\Gamma \left(M;T\left(M\right)\right)⋉\left(\Lambda^{0}\left(M\right)\right)⊕\Lambda^{3}\left(M\right))$ is determined by the following Lie commutator relationships:*

(28) $$\begin{array}{c} \left[\left(a_{1};r_{1},s_{1}\right),\left(a_{2};r_{2},s_{2}\right)\right]=(\left[a_{1},a_{2}\right];\\ \langle a_{1}|\nabla r_{2}\rangle -\langle a_{2}|\nabla r_{1}\rangle ,\langle \nabla |a_{1}s_{2}\rangle -\langle \nabla |a_{2}s_{1}\rangle ) \end{array}$$

*for any vector fields $a_{1},a_{2}\in diff_{0}\left(M\right)≃\Gamma \left(M;T\left(M\right)\right)\;$ and scalar quantities $r_{1},r_{2}\in \Lambda^{0}\left(M\right)\;$ and $s_{1},s_{2}\in \Lambda^{3}\left(M\right)$ on the manifold M.*

**Proof.**  Proof of the commutation relationships (28) easily follows from the group multiplication (14), if to take into account that tangent spaces $T\left(\Lambda^{0}\left(M\right)\right)≃\Lambda^{0}\left(M\right)$ and $T\left(\Lambda^{3}\left(M\right)\right)≃\left(\Lambda^{3}\left(M\right)\right).$ □

As an example, we calculate, for brevity, the Poisson bracket on the cotangent space $T_{\eta }^{*}\left(Diff\left(T^{n}\right)\right)$ at any $\eta \in Diff(T^{n}).$ Consider the cotangent space $T_{\eta }^{*}\left(Diff\left(T^{n}\right)\right)≃$$diff^{*}\left(T^{n}\right),$ the adjoint space to the tangent space $T_{\eta }\left(Diff\left(T^{n}\right)\right)$ of left invariant vector fields on $Diff(T^{n})$ at any $\eta \;\in \;Diff(T^{n}),\;$ and take the canonical symplectic structure on $\;T_{\eta }^{*}\left(Diff\left(T^{n}\right)\right)$ in the form $\omega^{\left(2\right)}\left(\mu ,\eta \right):=$δα(μ,η), where the canonical Liouville form α(μ,η)$:={\left(\mu |\delta \eta \right)}_{c}$$\in \Lambda_{\left(\mu ,\eta \right)}^{1}\left(T_{\eta }^{*}\left(Diff\left(T^{n}\right)\right)\right)$ at a point $\left(\mu ,\eta \right)\in T_{\eta }^{*}\left(Diff\left(T^{n}\right)\right)$ is defined *a priori* on the tangent space $T_{\eta }\left(Diff\left(T^{n}\right)\right)$ ≃Γ(T(M)) of right-invariant vector fields on the torus manifold $T^{n}.$ Having calculated the corresponding Poisson bracket of smooth functions ${\left(\mu |a\right)}_{c},{\left(\mu |b\right)}_{c}$$\in C^{\infty }\left(T_{\eta }^{*}\left(Diff\left(T^{n}\right)\right);R\right)$ on $T_{\eta }^{*}\left(Diff\left(T^{n}\right)\right)≃diff^{*}\left(T^{n}\right),$$\eta \in Diff(T^{n}),$ one can formulate the following proposition.

**Proposition** **3.** The Lie-Poisson bracket on the coadjoint space $T_{\eta }^{*}\left(Diff\left(T^{n}\right)\right)≃diff^{*}\left(T^{n}\right)$ is equal to the expression

(29) $$\left\{f,g\right\}\left(\mu \right)=\;{\left(\mu |\left[\delta f\left(\mu \right)/\delta \mu ,\delta g\left(\mu \right)/\delta \mu \right]\right)}_{c}$$

for any smooth functionals $f,g\in C^{\infty }\left(G^{*};R\right).$

**Proof.**  By definition [4,31] of the Poisson bracket of smooth functions $\;{\left(\mu |a\right)}_{c},{\left(\mu |b\right)}_{c}$$\in C^{\infty }\left(T_{\eta }^{*}\left(Diff\left(T^{n}\right)\right);R\right)\;$ on the symplectic space $T_{\eta }^{*}\left(Diff\left(T^{n}\right)\right),$ it is easy to calculate that

(30) $$\begin{array}{c} \left\{\mu \left(a\right),\mu \left(b\right)\right\}:=-\delta \alpha \left(X_{a},X_{b}\right)=\;\\ =-X_{a}{\left(\alpha |X_{b}\right)}_{c}+X_{b}{\left(\alpha |X_{a}\right)}_{c}+{\left(\alpha |\left[X_{a},X_{b}\right]\right)}_{c}, \end{array}$$

where $X_{a}:=\delta {\left(\mu |a\right)}_{c}/\delta \mu =a\in diff\left(T^{n}\right),X_{b}:=\delta {\left(\mu |b\right)}_{c}/\delta \mu =b\in diff\left(T^{n}\right).$ Since the expressions $X_{a}{\left(\alpha |X_{b}\right)}_{c}=0$ and $X_{b}{\left(\alpha |X_{a}\right)}_{c}=0$ owing the right-invariance of the vector fields $X_{a},X_{b}\in T_{\eta }\left(Diff\left(T^{n}\right)\right),$ the Poisson bracket (29) transforms into

(31) $$\begin{array}{c} \left\{{\left(\mu |a\right)}_{c},{\left(\mu |b\right)}_{c}\right\}={\left(\alpha |\left[X_{a},X_{b}\right]\right)}_{c}=\\ ={\left(\mu |\left[a,b\right]\right)}_{c}=\;{\left(\mu |\left[\delta {\left(\mu |a\right)}_{c}/\delta \mu ,\delta {\left(\mu |b\right)}_{c}/\delta \mu \right]\right)}_{c} \end{array}$$

for all (μ,η)∈$T_{\eta }^{*}\left(Diff\left(T^{n}\right)\right)≃diff^{*}\left(T^{n}\right),\eta \in Diff\left(T^{n}\right)$ and any $a,b\in diff(T^{n}).$ The Poisson bracket (29) is easily generalized to

(32) $${\left\{f,g\right\}\left(\mu \right)=\;\left(\mu |\left[\delta f\left(\mu \right)/\delta \mu ,\delta g\mu \right)/\delta \mu \right])}_{c}$$

for any smooth functionals $f,g\in C^{\infty }\left(G^{*};R\right),$ finishing the proof. □

Proceed now to the Grassmann algebra Λ(M) endowed with Hodge [37] star-isomorphism ∗:Λ(M)→Λ(M) subject to the usual metric on the tangent space T(M) and determine the adjoint space to the abelian subalgebra R(M)⊕Σ(M)≃$\Lambda^{0}\left(M\right)⊕\Lambda^{3}\left(M\right)$ as the space $*\Lambda^{3}\left(M\right)⊕*\Lambda^{0}\left(M\right)$ with respect to the following scalar product on Λ(M):

(33) $$\left(\alpha^{\left(n\right)}|\beta^{\left(m\right)}\right):=\delta_{mn}\int_{M}\left(\alpha^{\left(n\right)}\wedge *\beta^{\left(m\right)}\right)$$

for any $\alpha^{\left(n\right)},\beta^{\left(m\right)}\in \Lambda \left(M\right),m,n=\bar{0,3}.$ Then the adjoint space $G^{*},$ owing to the expressions (26) and (6), is described by means of the Eulerian variables $\left(\mu ;\rho d^{3}x,\sigma \right)\in G^{*}≃(\Lambda^{1}\left(M\right)⊗|\Lambda^{3}\left(M\right)\left|\right)⋉\left(\Lambda^{3}\left(M\right)⊕\Lambda^{0}\left(M\right)\right).$ The latter makes it possible to calculate the corresponding Lie-Poisson bracket on the adjoint space $G^{*}\;$ at a point $l:=\left(\mu ;\rho d^{3}x,\sigma \right)\in G^{*},$ generalizing the Poisson bracket (31):

(34) $$\begin{array}{c} \left\{f,g\right\}\left(l\right)={\left(l|\left[\delta f/\delta l,\delta g/\delta l\right]\right)}_{c}=\\ =\int_{M}d^{3}x\langle m\left|\left[\langle \frac{\delta f}{\delta m}\left|\nabla \right.\rangle \frac{\delta g}{\delta m}-\langle \frac{\delta g}{\delta m}\left|\nabla \right.\rangle \frac{\delta f}{\delta m}\right]\right.\rangle +\\ +\int_{M}\rho d^{3}x\left[\langle \frac{\delta f}{\delta m}\left|\nabla \frac{\delta g}{\delta \rho }\right.\rangle -\langle \frac{\delta g}{\delta m}\left|\nabla \frac{\delta f}{\delta \rho }\right.\rangle \;\right]+\\ +\int_{M}\sigma \left[\langle \nabla \left|\frac{\delta f}{\delta m}\frac{\delta g}{\delta \sigma }\right.\rangle -\langle \nabla \left|\frac{\delta g}{\delta m}\frac{\delta f}{\delta \sigma }\right.\rangle \right]d^{3}x \end{array}$$

for any smooth functionals $f,g\in C^{\infty }\left(G^{*};R\right),$ where we put, by definition, μ(x):=$\langle m\left(x\right)|dx\rangle ⊗d^{3}x,$$m\left(x\right)=\rho \left(x\right)v\left(x\right)\in T^{*}\left(M\right)$ for all x∈M and any t∈R.

Return now to the constraint (11) in the following variational form:

(35) $$\;\delta e_{t}\left(\rho_{t},\sigma_{t}\right)/\delta t=T_{t}\left(x_{t}\right)\delta \sigma_{t}\left(x_{t}\right)/\delta t+p_{t}^{\left(0\right)}\left(x_{t}\right)\rho_{t}^{-2}\left(x_{t}\right)\delta \rho_{t}\left(x_{t}\right)/\delta t,$$

which should hold at any $x_{t}\in M$ for all t∈R. Insomuch as, owing to the Euler Equation (10), the full (convective) derivative $\;\delta \sigma_{t}\left(x_{t}\right)/\delta t=0$ at any $x_{t}\in M$ for all t∈R, one checks once more that the expression (16) holds at any $x_{t}\in M$ for all t∈R. To determine the energy density function (16), we consider the Euler Equation (10) and check their Hamiltonian structure subject to the Poisson bracket (34), i.e., the existence of a Hamiltonian functional H: $G^{*}\to R,$ for which

(36) $$\frac{\partial }{\partial t}{\left(m;\rho ,\sigma \right)}^{⊺}=\left\{H,{\left(m,\rho ,\sigma \right)}^{⊺}\right\}$$

at any element $l={\left(m:=\rho v;\rho ,\sigma \right)}^{⊺}\in$$G^{*}.$ By means of easy calculations one obtains from the system (36) the variational gradient vector

(37) $$\delta H\left(l\right)/\delta l=(m\rho^{-1};-|m{|}^{2}/\left(2\rho^{2}\right)+w^{\left(0\right)}\;\left(\rho ,\sigma \right)+\rho \partial w^{\left(0\right)}\left(\rho ,\sigma \right)/\partial \rho ,\rho \partial w^{\left(0\right)}\left(\rho ,\sigma \right)/\partial \sigma ),$$

from which one derives [38,39,40] via the Volterra homotopy mapping

(38) $$H=\int_{0}^{1}{\left(\delta H\left(\lambda l\right)/\delta l|l\right)}_{c}d\lambda$$

the exact Hamiltonian expression

(39) $$H=\int_{M}{\left(|m\right|}^{2}/\left(2\rho \right)+\rho w^{\left(0\right)}\left(\rho ,\sigma \right)]d^{3}x,$$

coinciding with the expression (19) at $\;c\left(\sigma \right):={\left|m\right|}^{2}/\left(2\rho^{2}\right)={\left|v\right|}^{2}/2,$ as m:=ρv for v∈T(M). Thus, we obtain the internal energy density functional (16) as

(40) $$e_{t}\left(\rho_{t},\sigma_{t}\right)={\left|v_{t}\right|}^{2}/2+w_{t}^{\left(0\right)}\left(\rho_{t},\sigma_{t}\right),\;$$

for all $\rho :=\rho_{t}\in R\left(M\right),\sigma :=\sigma_{t}\in \Sigma \left(M\right)$ and $v_{t}\in T\left(M\right),$ satisfying simultaneously both the constraint (11) and the Euler evolution Equation (10) for all t∈R. Moreover, from the condition (17) one easily finds [33] the following important local differential relationship:

(41) $$\begin{array}{c} \partial \left[\rho_{t}\left(x_{t}\right)e_{t}\left(\rho_{t},\sigma_{t}\right)\right]/\partial t+\langle \nabla |\rho_{t}\left(x_{t}\right)v_{t}\left(x_{t}\right)(e_{t}\left(\rho_{t},\sigma_{t}\right)+\\ +\rho_{t}\left(x_{t}\right)\partial w_{t}^{\left(0\right)}\left(\rho_{t},\sigma_{t}\right)/\partial \rho_{t}\;)\rangle =0, \end{array}$$

satisfied for all $x_{t}\in M$ and t∈R, also confirming the energy conservation (39).

## 5. Hamiltonian Analysis: The Isothermal Liquid Dynamics

Consider a liquid motion governed by the following Euler equations governed by the Euler equations

(42) $$\begin{array}{c} \partial v/\partial t+\langle v|\nabla \rangle v+\rho^{-1}\nabla p^{\left(0\right)}=0,\\ \partial \rho /\partial t+\langle \nabla |\rho v\rangle =0,\partial T/\partial t+\langle v|\nabla T\rangle =0, \end{array}$$

and describing the ideal compressible and isothermal motion of an ideal compressible and adiabatic fluid in a spatial domain $M\subset R^{3},$ as the temperature $T_{t}\left(x_{t}\right)=T_{0}\left(x_{t}\right)$ at any evolution point $x_{t}:=\eta_{t}\left(X\right)\;\in M$ for all X∈M and t∈R. The evolution (42) is considered to be *a priori* thermodynamically quasi-stationary, i.e., the following, *infinitesimal convective* energy relationship

(43) $$\delta {\tilde{h}}_{t}\left(\rho_{t},T_{t}\right)\;=-\sigma_{t}\left(x_{t}\right)\;\delta T_{t}\;+p_{t}^{\left(0\right)}\left(x_{t}\right)\;\rho_{t}^{-2}\delta \rho_{t}\;$$

holds for all densities $\rho_{t}\in R\left(M\right),$ temperature $T_{t}$∈T(M) and specific entropy $\sigma_{t}\in \Sigma \left(M\right),$ where $\tilde{h}:R\left(M\right)\times T\left(M\right)\to R$ denotes the corresponding internal specific fluid “energy” and the variation sign “δ” means the change subject to both the temporal variable t∈R and the spatial variable $x_{t}\in M.$ Under the imposed *isothermal condition* $\delta T_{t}=0$ the expression (43) transforms into

(44) $${\tilde{h}}_{t}\left(\rho_{t},T_{t}\right)=\;{\left|v_{t}\right|}^{2}/2+{\tilde{w}}_{t}^{\left(0\right)}\left(\rho_{t},T_{t}\right),$$

where ${\tilde{w}}_{t}^{\left(0\right)}\left(\rho_{t},T_{t}\right):=w_{t}^{\left(0\right)}\left(\rho_{t},\sigma_{t}\right){|}_{\sigma_{t}:=\tilde{\sigma }\left(\rho_{t},T_{t}\right)}-T_{t}\sigma_{t}\left(\rho_{t},T_{t}\right),$ is the specific potential liquid energy for *the isothermal flow*, determined at $\sigma_{t}:=\sigma_{t}\left(\rho_{t},T_{t}\right),$ solving the functional relation $\;T_{t}=\partial w_{t}^{\left(0\right)}\left(\rho_{t},\sigma_{t}\right)/\partial \sigma_{t}$∈T(M) subject to the entropy argument $\sigma_{t}\in \Sigma \left(M\right),$ if the condition $\partial^{2}w_{t}^{\left(0\right)}\left(\rho_{t},\sigma_{t}\right)/\partial \sigma_{t}^{2}\neq 0$ holds for all densities $\rho_{t}\in R\left(M\right)\;$ and t∈R.

Observe now that the third equation of (42) is exactly equivalent to the internal average fluid kinetic energy conservation integral relationship

(45) $$\frac{d}{dt}\int_{D_{t}}\rho_{t}\left(x_{t}\right)T_{t}\left(x_{t}\right)d^{3}x_{t}=0$$

over the domain $D_{t}:=\eta_{t}\left(D\right)\subset M,$ where a smooth submanifold $D=D_{t}{|}_{t=0}$⊂M is chosen arbitrary and $\eta_{t}:$M→M,t∈R, denotes the corresponding evolution subgroup of the diffeomorphism group $Diff_{0}\left(M\right),$ generated by the Euler evolution Equation (42). The relationship (45) simply means that if the density function $\rho_{t}\in R\left(M\right)$ transforms under diffeomorphism group $Diff_{0}\left(M\right)$ action as the abelian functional group $R\left(M\right)≃\Lambda^{0}\left(M\right),$ the corresponding transformation of the temperature $T_{t}\in T\left(M\right)$ is induced by the diffeomorphism group $Diff_{0}\left(M\right)$ action on the related abelian group $T\left(M\right)≃\Lambda^{3}\left(M\right).$ Concerning the energy density (44) one easily obtains the following differential relationship:

(46) $$\partial \left[\rho_{t}\left(x_{t}\right){\tilde{h}}_{t}\left(\rho_{t},T_{t}\right)\right]/\partial t+\langle \nabla |\rho_{t}\left(x_{t}\right)v_{t}\left(x_{t}\right)\left[{\tilde{h}}_{t}\left(\rho_{t},T_{t}\right)\rangle +\rho_{t}\partial {\tilde{w}}_{t}^{\left(0\right)}\left(\rho_{t},T_{t}\right)/\partial \rho_{t}\right]\;\rangle =0,$$

satisfied for all t∈R. As a simple consequence of the relationship (46) one obtains that the following functional

(47) $$\tilde{H}=\int_{D_{t}}\rho_{t}\left(x_{t}\right){\tilde{h}}_{t}\left(\rho_{t},T_{t}\right)d^{3}x_{t}$$

is conserved over the domain $D_{t}:=\eta_{t}\left(D\right)\subset M,$t∈R, where a smooth submanifold $D=D_{t}{|}_{t=0}$⊂M is chosen arbitrary.

Similarly to reasonings of Section 3, one can construct now the differential-functional group space Diff(M)×(R(M)×T(M)) and formulate the following easily checkable proposition.

**Proposition** **4.** *The differential-functional group functional manifold Diff(M)×(R(M)×T(M)) in Eulerian coordinates is a smooth Banach group G:=Diff(M)⋉(R(M)×T(M)), equal to the semidirect product of the diffeomorphism group Diff(M) and the direct product R(M)×T(M) of abelian functional $R\left(M\right)≃\Lambda^{0}\left(M\right)\;$ and density $\;\;T\left(M\right)≃\Lambda^{3}\left(M\right)\;$ groups, endowed with the following group multiplication law:*

(48) $$\begin{array}{c} \left(\varphi_{1};r_{1},\tau_{1}d^{3}x^{\;\;}\right)\circ \left(\varphi_{2};r_{2},\tau_{2}d^{3}x\right)=\\ +(\varphi_{2}\cdot \varphi_{1};r_{1}+r_{2}\cdot \varphi_{1},\tau_{1}d^{3}x+\left(\tau_{2}d^{3}x\right)\cdot \varphi_{1}) \end{array}$$

*for arbitrary elements $\varphi_{1},\varphi_{2}\in Diff\left(M\right),r_{1},r_{2}\in \Lambda^{0}\left(M\right)$ and $\;\tau_{1}d^{3}x,\tau_{2}d^{3}x\in \Lambda^{3}\left(M\right).$*

This proposition allows a simple enough interpretation, namely, it means that the adiabatic mixing of the $G∋(\varphi_{2};r_{2},\tau_{2}d^{3}x)\;$ liquid configuration with the $G∋(\varphi_{1};r_{1},\;\tau_{1}d^{3}x)$ liquid configuration amounts to summation of their spatially shifted densities, simultaneously changing the common specific kinetic energy, proportional [41,42,43] to the liquid temperature, owing to the fact that some space of the domain *M* is already occupied by the first liquid configuration and the second one should be diffeomorphically shifted from this configuration to another free part of the spatial domain M with fixed and bounded volume. The diffeomorphism group Diff(M) can be naturally restricted to the factor-group $Diff_{0}\left(M\right):=Diff\left(M\right)/Diff_{\rho_{0},T_{0}}\left(M\right)$ subject to the stationary normal symmetry subgroup $\;Diff_{0}\left(M\right):=Diff_{\rho_{0},T_{0}}\left(M\right)\subset Diff\left(M\right),$ where

(49) $$Diff_{\rho_{0},T_{0}}\left(M\right):=\left\{\varphi \in Diff\left(M\right):\rho_{0}\left(X\right)=J_{\varphi (X)}\rho_{0}\left(\varphi \left(X\right)\right),T_{0}\left(X\right)=T_{0}\left(\varphi \left(X\right)\right)\right\}$$

for any X∈M. Based on the construction above one can proceed to studying the extended Banach group $G:=Diff_{0}\left(M\right)⋉\left(\Lambda^{0}\left(M\right)\times \Lambda^{3}\left(M\right)\right)$ action on the cotangent bundle $T_{g_{\eta }}^{*}\left(G\right)$ at $g_{\eta }:=\left(\eta ;\rho_{0},T_{0}\right)\in G_{0},$ generated by the fluid evolution with respect to the Euler Equation (42). The related fluid motion is naturally modelled by means of the coadjoint action of the corresponding Lie algebra $G≃T_{g_{\eta }}\left(G_{0}\right)≃$$\Gamma \left(M;T\left(M\right)\right)⋉\left(\Lambda^{0}\left(M\right)⊕\Lambda^{3}\left(M\right)\right)$ of the group $G_{0},$$g_{\eta }=Id\in G_{0},\;\;$ on its adjoint space $G^{*}≃\left(\Lambda^{1}\left(M\right)⊗\Lambda^{3}\left(M\right)\right)⋉\left(*\Lambda^{0}\left(M\right)⊕*\Lambda^{3}\left(M\right)\right)$$=(\Lambda^{1}\left(M\right)⊗\Lambda^{3}\left(M\right))$$⋉(\Lambda^{3}\left(M\right)⊕\Lambda^{0}\left(M\right)).$

The related Lie structure on G easily ensues from the action (48):

(50) $$\begin{array}{c} \left[\left(a_{1};r_{1},\tau_{1}\right),\left(a_{2};r_{2},\tau_{2}\right)\right]=(\left[a_{1},a_{2}\right];\;\\ \langle a_{1}|\nabla r_{2}\rangle -\langle a_{2}|\nabla r_{1}\rangle ,\langle \nabla |a_{1}\tau_{2}\rangle -\langle \nabla |a_{2}\tau_{1}\rangle ) \end{array}$$

for any representative elements $\left(a_{1};r_{1},\tau_{1}\right)$ and $(a_{2};r_{2},\tau_{2})\in G.$ Moreover, as the cotangent bundle $T_{g_{\eta }}^{*}\left(G_{0}\right)$ at $g_{\eta }=Id\in G_{0}\;$ is diffeomorphic to the adjoint space $G^{*}$ to the Lie algebra G of the Banach group $G_{0},$ it is *a priori* endowed with the canonical Lie-Poisson structure

(51) $$\begin{array}{c} \left\{f,g\right\}\left(l\right)={\left(l|\left[\delta g/\delta l,\delta f/\delta l\right]\right)}_{c}=\;\\ =\int_{M}d^{3}x\langle m\left|\left[\langle \frac{\delta f}{\delta m}\left|\nabla \right.\rangle \frac{\delta g}{\delta m}-\langle \frac{\delta g}{\delta m}\left|\nabla \right.\rangle \frac{\delta f}{\delta m}\right]\right.\rangle +\;\\ +\int_{M}\rho d^{3}x\left[\langle \frac{\delta f}{\delta m}\left|\nabla \frac{\delta g}{\delta \rho }\right.\rangle -\langle \frac{\delta g}{\delta m}\left|\nabla \frac{\delta f}{\delta \rho }\right.\rangle \;\right]+\;\\ +\int_{M}T\left[\langle \nabla \left|\frac{\delta f}{\delta m}\frac{\delta g}{\delta T}\right.\rangle -\langle \nabla \left|\frac{\delta g}{\delta m}\frac{\delta f}{\delta T}\right.\rangle \right]d^{3}x \end{array}$$

for any smooth functional $f,g\in C^{\infty }\left(G^{*};R\right),$ where we put, by definition, an element $l:=\left(m;\rho ,T\right)≃\left(\mu ;\rho d^{3}x,T\right)\in G^{*},$μ(x):=$\langle m\left(x\right)|dx\rangle ⊗d^{3}x,$$m\left(x\right)=\rho \left(x\right)v\left(x\right)\in T^{*}\left(M\right)$ for all x∈M and t∈R, one can easily check that the flow (42) is Hamiltonian:

(52) $$dl/dt=\{\tilde{H},l\}$$

subject to the adjusted Hamiltonian functional (47):

(53) $$\tilde{H}:=\int_{M}h_{t}\left(\rho_{t},T_{t}\right)d^{3}x_{t}=\int_{M}\rho_{t}\left(\right|m_{t}{|}^{2}/2\rho_{t}^{2}+{\tilde{w}}_{t}^{\left(0\right)}\left(\rho_{t},T_{t}\right))d^{3}x.$$

satisfying the conservative condition $d\tilde{H}/dt$=0 for all t∈R, following simultaneously both from (52) and from the differential relationship (46).

## 6. Hamiltonian Analysis: The Adiabatic Magneto-Hydrodynamic Superfluid Motion

### 6.1. Geometric Description

We start with considering a quasi-neutral superfluid contained in a domain $M\subset R^{3}$ and interacting with a “frozen” sourceless magnetic field $B\in B\left(M\right)\subset C^{\infty }\left(M;E^{3}\right),$ satisfying the superconductivity conditions

(54) $$\tilde{E}:=E+v\times B=0,\;\partial E/\partial t=\nabla \times B,$$

where $\tilde{E}:M\to E^{3}$ is the internal net superfluid electric field, $E=-\partial A/\partial t:M\to E^{3}$ and $B=\nabla \times A:M\to E^{3}$ are the internal electric and magnetic fields, respectively, generated by the corresponding magnetic vector field potential $A:M\to E^{3},$v:M⟶T(M) is the superfluid velocity and “×” denotes the usual vector product in the Euclidean space $E^{3}.$ The following natural boundary conditions ${\langle n|v\rangle |}_{\partial M}=0$ and ${\langle n|B\rangle |}_{\partial M}=0$ are imposed on the superfluid flow, where $n\in T^{*}\left(M\right)$ is the vector normal to the boundary ∂M, which is considered to be almost everywhere smooth.

Then in adiabatic magnetohydrodynamics (MHD) quasi-neutral superconductive superfluid motion is described by the following system of evolution equations:

(55) $$\begin{array}{c} \partial v/\partial t+\langle v|\nabla \rangle v+\rho^{-1}\nabla p-\rho^{-1}\left(\nabla \times B\right)\times B=0,\;\\ \partial \rho /\partial t+\langle \nabla |\rho v\rangle =0,\partial \sigma /\partial t+\langle u|\nabla \sigma \rangle =0,\partial B/\partial t=\nabla \times (v\times B), \end{array}$$

where, as before, $\rho :=\rho_{t}$∈R(M) is the superfluid density, $B:=B_{t}:M⟶E^{3}$ is the “frozen” into the superfluid magnetic field, $p:=p_{t}:M⟶R$ is the internal liquid pressure and $\sigma :=\sigma_{t}$:M⟶R is the specific superfluid entropy at time t∈R. The latter is related with the internal MHD superfluid specific energy function $e=e_{t}\left(\rho_{t},\sigma_{t}\right)$ owing to the first thermodynamic law:

(56) $$T_{t}\left(\rho_{t},\sigma_{t}\right)\;\delta \sigma_{t}=\delta e_{t}\left(\rho_{t},\sigma_{t}\right)-p_{t}\rho_{t}^{-2}\delta \rho_{t},$$

satisfied for any admissible variations of the phase space parameters $\rho_{t}\in R\left(M\right),$$\sigma_{t}\in \Sigma \left(M\right),$ where $T_{t}=T_{t}\left(\rho_{t},\sigma_{t}\right)$ is the internal absolute temperature in the superfluid for t∈R. The isentropic condition $\delta \sigma_{t}\left(x_{t}\right)=0,$ where $x_{t}:=\eta_{t}\left(X\right)\in M$ for all X∈M and the related to (55) evolution diffeomorphism $\eta_{t}\in Diff\left(M\right),t\in R,$ entails the following expression for the specific internal energy

(57) $$e_{t}\left(\rho_{t},\sigma_{t}\right)=\;w_{t}^{\left(0\right)}\left(\rho_{t},\sigma_{t}\right)+c_{t}\left(\rho_{t},B_{t}\right),$$

where $w_{t}^{\left(0\right)}:R\left(M\right)\times \Sigma \left(M\right)\to C^{\infty }\left(M;R\right)$ is the corresponding internal potential specific energy density and $c_{t}:R\left(M\right)\times B\left(M\right)\to C^{\infty }\left(M;R\right)$ is some still unknown function, depending in general on the imposed magnetic field $B_{t}:M⟶E^{3},t\in R.$

Let us now analyze, as before, the mathematical structure of quantities $\left(\rho_{t},\sigma_{t},B_{t}\right)\in R\left(M\right)\times \Sigma \left(M\right)\times B\left(M\right)$ as the *physical observables* subject to their evolution (55) with respect to the group diffeomorphisms $\eta_{t}\in Diff\left(M\right),t\in R,$ generated by the liquid motion vector field $dx_{t}/dt=v_{t}\left(x_{t}\right),x_{t}:=\eta_{t}\left(X\right),t\in R,X\in M:$

(58) $$\begin{array}{c} L_{d/dt}\left(\langle \rho_{t}v_{t}|dx_{t}\rangle d^{3}x_{t}\right)=[-dp_{t}^{\left(0\right)}+\rho_{t}^{-1}d|v_{t}{|}^{2}/2+\langle B_{t}|\nabla \rangle \langle B_{t}|dx_{t}\rangle )]\rho_{t}d^{3}x_{t},\;\\ L_{d/dt}\left(\rho_{t}d^{3}x_{t}\right)=0,\;\;\;L_{d/dt}\sigma_{t}=0,\;\;\;L_{d/dt}\left(*\langle B_{t}|dx_{t}\rangle \right)=0, \end{array}$$

where $L_{d/dt}:\Lambda \left(M\right)\to \Lambda \left(M\right)$ denotes the corresponding Lie derivative with respect to the vector field $d/dt:=\partial /\partial t+\langle v_{t}|\nabla \rangle \in \Gamma \left(M\times R;T\left(M\right)\right),t\in R.$ The relationships (58) mean that the space of physical observables, being by definition, the adjoint space $G_{em}^{*}:=\;\Lambda^{1}\left(M\right)d^{3}x\times \left(\Lambda^{3}\left(M\right)⊕\Lambda^{0}\left(M\right)⊕\Lambda^{2}\left(M\right)\right)$ to the extended configuration space is equal to $G_{em}:=diff\left(M\right)\times \left(\Lambda^{0}\left(M\right)⊕\Lambda^{3}\left(M\right)⊕\Lambda^{1}\left(M\right)\right)$$≃T_{Id}\left(G_{em}\right),$ the tangent space at the identity Id to the extended differential-functional group manifold $\;G_{em}:=Diff\left(M\right)\times \Lambda^{0}\left(M\right)$$\times \Lambda^{3}\left(M\right)\times \Lambda^{1}\left(M\right)≃Diff\left(M\right)\times R\left(M\right)$×Σ(M)×B(M), where we have naturally identified the abelian group product $\Lambda^{0}\left(M\right)$$\times \Lambda^{3}\left(M\right)\times \Lambda^{1}\left(M\right)$ with its direct tangent space sum $T(\Lambda^{0}\left(M\right))$$⊕T(\Lambda^{3}\left(M\right))$$⊕T(\Lambda^{1}\left(M\right)).$

Consider now the constructed differential-functional group manifold $\;G_{em}$ in Eulerian variables, on which one naturally acts the Diff(M)-group $\;Diff\left(M\right)\times G_{em}\to G_{em}$ the standard way:

(59) $$\begin{array}{c} (\eta \circ \varphi )(X):=\varphi (\eta (X)),(\eta \circ r)(X):=r(\eta (X)),\;\\ \eta \circ \;\left(s\left(X\right)d^{3}X\;\right):=\;\eta^{*}\left(s\left(X\right)d^{3}X\;\right),\;\\ \eta \circ \;\langle b\left(X\right)|dX\rangle \;:=\;\eta^{*}\langle b\left(X\right)|d^{3}X\rangle \end{array}$$

for η∈Diff(M),X∈M and any (φ;r,s,b)∈Diff(M)×R(M)×Σ(M)×B(M). Then, taking into account the suitably extended action (59) on the differential-functional manifold $G_{em},$ one can formulate the following easily checkable and crucial for what will follow further proposition.

**Proposition** **5.** *The differential-functional group manifold $\;G_{em}:=Diff\left(M\right)\times R\left(M\right)\times$Σ(M)×B(M) in Eulerian coordinates is a smooth symmetry Banach group $G_{em}:=Diff\left(M\right)⋉\left(R\left(M\right)\times \Sigma \left(M\right)\times B\left(M\right)\right),$ equal to the semidirect product of the diffeomorphism group Diff(M) and the direct product R(M)×Σ(M)×B(M) of abelian functional $R\left(M\right)≃\Lambda^{0}\left(M\right),$ density $\;\;\Sigma \left(M\right)≃\Lambda^{3}\left(M\right)$ and one-form $\;\;B\left(M\right)\;≃\Lambda^{1}\left(M\right)$ groups, endowed with the following group multiplication law in Eulerian variables:*

(60) $$\begin{array}{c} \left(\varphi_{1};r_{1},s_{1}d^{3}x,\langle b_{1}|dx\rangle \right)\circ \left(\varphi_{2};r_{2},s_{2}d^{3}x,\langle b_{2}|dx\rangle \right)=\;\\ =(\varphi_{2}\cdot \varphi_{1};r_{1}+r_{2}\cdot \varphi_{1},s_{1}d^{3}x+\left(s_{2}d^{3}x\right)\cdot \varphi_{1},\langle b_{1}|dx\rangle +\langle b_{2}|dx\rangle \circ \varphi_{1}) \end{array}$$

*for arbitrary elements $\varphi_{1},\varphi_{2}\in Diff\left(M\right),r_{1},r_{2}\in \Lambda^{0}\left(M\right),\;s_{1}d^{3}x,s_{2}d^{3}x$$\in \Lambda^{3}\left(M\right)\;$ and $\langle b_{1}|dx\rangle ,$$\langle b_{2}|dx\rangle \in \Lambda^{1}\left(M\right).$*

Thus, one can proceed to studying the corresponding coadjoint action of the Lie algebra $G_{em}≃T_{Id}\left(G_{em}\right),$$Id\in G_{em},$ on the adjoint space $G_{em}^{*}.$ As the Lagrangian configuration $\eta_{0}\in Diff\left(M\right)$ and the entropy $\sigma_{0}\in \Sigma \left(M\right)$ are assumed to be invariant under the Banach diffeomorphism group action Diff(M), the resulting group action can be reduced to the factor-group $\;Diff_{0}\left(M\right):=Diff\left(M\right)/Diff_{\eta_{0},\sigma_{0}}\left(M\right)$ action on the semidirect group product $G_{em,0}:=Diff_{0}\left(M\right)⋉\left(R(M)\times \Sigma (M)\times B(M\right).$ Based on the multiplication law (60) one easily calculates the following Lie algebra commutation relationships:

(61) $$\begin{array}{c} \left[\left(a_{1};r_{1},s_{1},b_{1}\right),\left(a_{2};r_{2},s_{2},b_{2}\right)\right]=(\left[a_{1},a_{2}\right];\langle a_{1}|\nabla r_{2}\rangle -\;\\ -\langle a_{2}|\nabla r\rangle ,\langle \nabla |a_{1}b_{2}\rangle -\langle \nabla |a_{2}s_{1}\rangle ,\langle a_{1}|\nabla \rangle b_{2}-\;\\ -\langle a_{2}|\nabla \rangle b_{1}+\langle b_{2}|\circ \nabla a_{1}\rangle -\langle b_{1}|\circ \nabla a_{2}\rangle ) \end{array}$$

for any elements $a_{1},a_{2}\in diff\left(M\right)≃T\left(M\right),r_{1},r_{2}\in R\left(M\right)≃\Lambda^{0}\left(M\right),$$s_{1},s_{2}\in \Sigma \left(M\right)≃\Lambda^{3}\left(M\right)$ and $b_{1},b_{2}\in B\left(M\right)≃\Lambda^{1}\left(M\right).$

The adjoint space to the semidirect product Lie algebra $G_{em,0}=diff\left(M\right)⋉(R\left(M\right)⊕\Sigma \left(M\right)$⊕B(M)) can be, naturally, written symbolically as the space $G_{em,0}^{*}=\left(\Lambda^{1}\left(M\right)⊗\Lambda^{3}\left(M\right)\right)\times (*\Lambda^{0}\left(M\right)⊕*\Lambda^{3}\left(M\right)$$⊕*\Lambda^{1}\left(M\right))$$\;=diff^{*}\left(M\right)\times (\Lambda^{3}\left(M\right)⊕$$\Lambda^{0}\left(M\right)⊕\Lambda^{2}\left(M\right)),$ where as before, the mapping ∗:Λ(M)→Λ(M) denotes the Hodge isomorphism. Then, taking into account the adjoint space $G_{em,0}^{*}$ to the Lie algebra $G_{em,0}$ is endowed with the following [5,6,19,33,44,45] canonical Lie-Poisson bracket

(62) $$\begin{array}{c} \left\{f,g\right\}:=\int_{M}\langle m|\langle \frac{\delta f}{\delta m}|\nabla \rangle \frac{\delta g}{\delta m}-\langle \frac{\delta g}{\delta m}|\nabla \rangle \frac{\delta f}{\delta m}\rangle d^{3}x+\\ +\int_{M}\rho \left(\langle \frac{\delta f}{\delta m}|\nabla \frac{\delta g}{\delta \rho }\rangle -\langle \frac{\delta g}{\delta m}|\nabla \frac{\delta f}{\delta \rho }\rangle \right)d^{3}x+\int_{M}\sigma \langle \nabla |\left(\frac{\delta f}{\delta m}\frac{\delta g}{\delta \sigma }-\frac{\delta g}{\delta m}\frac{\delta f}{\delta \sigma }\right)\rangle d^{3}x+\\ +\int_{M}\left(\langle B|\langle \frac{\delta f}{\delta m}|\nabla \rangle \frac{\delta g}{\delta B}-\langle \frac{\delta g}{\delta m}|\nabla \rangle \frac{\delta f}{\delta B}\rangle +\langle \frac{\delta f}{\delta B},\langle B|\nabla \rangle \frac{\delta g}{\delta m}\rangle -\langle \frac{\delta g}{\delta B},\langle B|\nabla \rangle \frac{\delta f}{\delta m}\rangle \right)d^{3}x\; \end{array}$$

for any smooth functionals $f,g\in D(G_{em,0}^{*})$ on the adjoint space $G^{*},$ where, as before, we denoted by $m:=\rho v\in T^{*}\left(M\right)$ the specific momentum of the superfluid. The bracket (62) naturally ensues from the canonical symplectic structure on the cotangent phase space $T^{*}\left(G_{em,0}\right),$ as it was before demonstrated in Section 4.

Write down now the first two equations of the Euler MHD system (55) as the local fluid mass and momentum conservation laws in the integral Ampere–Newton form

(63) $$\begin{array}{c} \frac{d}{dt}\int_{D_{t}}\rho_{t}d^{3}x_{t}=0,\;\;\;\frac{d}{dt}\int_{D_{t}}\rho_{t}v_{t}\;d^{3}x_{t}+\\ +\int_{\partial D_{t}}p_{t}^{\left(0\right)}\left(x_{t}\right)d^{2}S_{t}-\int_{D_{t}}\langle B_{t}\left(x_{t}\right)|\nabla \rangle B_{t}\left(x_{t}\right)d^{3}x_{t}=0, \end{array}$$

which is completely equivalent to the relationships (58) and where $p_{t}^{\left(0\right)}\;:M\to R_{+}$ is the net internal superfluid pressure, $\left(\nabla \times B_{t}\left(x_{t}\right)\right)\times B_{t}\left(x_{t}\right):$$M\to C^{\infty }\left(M;E^{3}\right)$ is the spatially distributed Lorentz force on the superfluid, $\;d^{2}S_{t}$ is the respectively oriented surface measure on the boundary $\partial D_{t}$ for the domain $D_{t}:=\eta_{t}(D$)⊂M,t∈R, and a smooth submanifold *D*⊂M is chosen arbitrary. Taking into account that $\left(\nabla \times B_{t}\left(x_{t}\right)\right)\times B_{t}\left(x_{t}\right)=\langle B_{t}|\nabla \rangle B_{t}-\nabla \langle B_{t}|B_{t}\rangle /2$ for any $B_{t}\in B\left(M\right),$ the second integral relationship (63) becomes equivalent to the following:

(64) $$\partial v_{t}/\partial t+\langle v_{t}|\nabla \rangle v_{t}+\rho_{t}^{-1}\nabla p_{t}^{\left(0\right)}\left(\rho_{t},\sigma_{t}\right)-\rho_{t}^{-1}\langle B_{t}|\nabla \rangle B_{t}=0,$$

where we have represented the internal superfluid pressure quantity as

(65) $$p_{t}\left(x_{t}\right):=p_{t}^{\left(0\right)}\left(\rho_{t},\sigma_{t}\right)-\langle B_{t}|B_{t}\rangle /2$$

for some mapping $p_{t}^{\left(0\right)}:R\left(M\right)\times \Sigma \left(M\right)\to C^{\infty }\left(M;R\right),$ strictly depending only on the internal liquid configuration $\eta_{t}\in Diff\left(M\right)\;$ for all t∈R.

Based on the Poisson bracket expression (62), one can now easily determine the Hamiltonian function H:M→R, corresponding to the Euler evolution Equation (55) on the adjoint space $G^{*}:$

(66) $$\begin{array}{c} H=\int_{M}\rho_{t}\left(\right|m_{t}{|}^{2}/\left(2\rho_{t}^{2}\right)+\;w_{t}^{\left(0\right)}\left(\rho_{t},\sigma_{t}\right)+\\ +|B_{t}{|}^{2}/\left(2\rho_{t}\right))dx_{t}^{3}:=\int_{M}\rho \left(x_{t}\right)e_{t}\left(\rho_{t},\sigma_{t}\right)d^{3}x_{t}, \end{array}$$

where the quantity

(67) $$\begin{array}{ccc} e_{t}\left(\rho_{t},\sigma_{t}\right) & = & |m_{t}{|}^{2}/\left(2\rho_{t}^{2}\right)+\;w_{t}^{\left(0\right)}\left(\rho_{t},\sigma_{t}\right)+\\ +|B_{t}{|}^{2}/\left(2\rho_{t}\right) & : & =|m_{t}{|}^{2}/\left(2\rho_{t}^{2}\right)+\;w_{t}\left(\rho_{t},\sigma_{t}\right) \end{array}$$

denotes the specific internal superfluid energy, modified by means of the “frozen” magnetic field $B_{t}\in B\left(M\right),t\in R,$ replacing the before defined in Section 3 internal specified potential energy $w_{t}^{\left(0\right)}\left(\rho_{t},\sigma_{t}\right)$ by the shifted specified potential energy quantity $\;w_{t}\left(\rho_{t},\sigma_{t}\right):=$$w_{t}^{\left(0\right)}\left(\rho_{t},\sigma_{t}\right)$$+|B_{t}{|}^{2}/\left(2\rho_{t}\right).$ In particular, the Equation (64) reduces to the equivalent Hamilton expression

(68) $$\partial m_{t}/\partial t=\left\{H,m_{t}\right\}$$

for $m_{t}\in T^{*}\left(M\right)≃diff^{*}\left(M\right)$ and all t∈R. It is also seen that if $B_{t}\to 0$ uniformly with respect to time t∈R, the internal energy expression (67) brings about that (40). Recall now that the following quasi-stationary second thermodynamic energy conservation law

(69) $$\delta e_{t}\left(\rho_{t},\sigma_{t}\right)=\rho_{t}^{-2}p_{t}\left(x_{t}\right)\delta \rho_{t}+T_{t}\left(x_{t}\right)\delta \sigma_{t}$$

holds for all admitted superfluid variations $\delta \rho_{t}\in R\left(M\right)$ and $\delta \sigma_{t}\in \Sigma \left(M\right),t\in R.$ As, by isentropic assumption, $\delta \sigma_{t}=0\;$ for all t∈R along fluid streamlines, for the internal pressure one easily obtains the expression $p_{t}\left(x_{t}\right)=\rho_{t}^{2}\partial w_{t}^{\left(0\right)}\left(\rho_{t},\sigma_{t}\right)/\partial \rho_{t}-\langle B_{t}|B_{t}\rangle /2,$ exactly coinciding with that of (65).

The Hamiltonian function (66) satisfies evidently the conservation condition dH/dt=0 for all t∈R. To check this directly, it is enough to observe [33] that the following differential relationship

(70) $$\partial e_{t}\left(\rho_{t},\sigma_{t}\right)/\partial t+\langle \nabla |\rho_{t}v_{t}\left[e_{t}\left(\rho_{t},\sigma_{t}\right)+\rho_{t}\partial w_{0}\left(\rho_{t},\sigma_{t}\right)/\partial \rho_{t}-{\left|B_{t}\right|}^{2}/2\right])=0$$

holds for all t∈R and whose integration over the domain $M\subset R^{3}$ easily gives rise to the conservation of the Hamiltonian function (66).

### 6.2. Magneto-Hydrodynamic Invariants and Their Geometry

The importance of spatial invariants describing the stability [33] of MHD superfluid motion was previously stated long ago [32,33,36,46]. Based on the modern symplectic theory of differential–geometric structures on manifolds, we devise a unified approach to study MHD invariants of compressible superfluid flow, related with specially constructed symmetry structures and commuting to each other vector fields on the phase space.

We start from a useful differential-geometric observation that the magneto-hydrodynamic Euler equations Γ(M;T(M)) action on the adjoint space to the Lie algebra G of the Banach group $G=Diff\left(M\right)⋉(\Lambda^{0}\left(M\right)⊕\Lambda^{3}\left(M\right)⊕{*}^{1}\left(M\right)),$ generated by the following vector field differential relationship:

(71) $$dx_{t}/dt=v_{t}\left(x_{t}\right),$$

where $x_{t}=\eta_{t}\left(X\right)\in M,$X∈M, and $v_{t}:M\to T\left(M\right),$t∈R, is an acceptable time-dependent vector field on the domain M, describing the adiabatic superfluid and superconductive motion via the diffeomorphism subgroup mappings $\eta_{t}\in Diff\left(M\right),\eta_{t}{|}_{t=0}=$$\eta_{0}\in Diff_{0}\left(M\right).$ Taking into account that the initial superfluid configuration $\eta_{0}\in Diff\left(M\right)$ is fixed, one can define, following reasonings from [47], a new differential relationship

(72) $$dx_{\tau }/dt=u_{t}\left(x_{\tau }\right)\;$$

on the domain *M* with respect to the evolution variable τ∈R, parameterized by the time parameter t∈R, where $u_{t}:M\to T\left(M\right),$ is a τ-independent vector field on M, generating the diffeomorphism subgroup $\psi_{t}\in Diff\left(M\right),$$x_{\tau }:=\psi_{\iota }\left(\eta_{0}\left(X\right)\right),X\in M,$ commuting to that generated by the vector field (71), i.e., $\eta_{t}\circ \psi_{\iota }=\psi_{t}\circ \eta_{\iota }$ for all t,τ∈R. The action of the diffeomorphism subgroup $\psi_{t}\in Diff\left(M\right)$ at any fixed time t∈R can be naturally interpreted as rearranging the particle configurations in the superfluid not changing its other dynamic characteristics. If to denote the corresponding Lie derivatives with respect to the vector fields (71) and (72) by differential expressions $L_{d/dt}:=\partial /\partial t+\langle v_{t}|\nabla \rangle \circ :C^{\infty }\left(M;R\right)\to C^{\infty }\left(M;R\right)$ and $L_{u_{t}}:=\langle u_{t}|\nabla \rangle \circ :$$C^{\infty }\left(M;R\right)\to C^{\infty }\left(M;R\right),$ the commutation condition $\eta_{t}\circ \psi_{\iota }=\psi_{t}\circ \eta_{\iota }$ for all t,τ∈R is equivalently rewritten as the operator commutator

(73) $$[L_{d/dt},L_{u_{t}}]=0.$$

Consider now an arbitrary integral invariant of the MHD superfluid, governed by the Euler system (55):

(74) $$I=\int_{D_{t}}\rho_{t}\left(x_{t}\right)\gamma_{t}\left(m_{t};\rho_{t},\sigma_{t},B_{t}\right)d^{3}x_{t},$$

generated by some specific density functional $\gamma_{t}:G^{*}\to C^{\infty }\left(M\times R;R\right)$ and held over the domain $D_{t}=\eta_{t}\left(D\right)$ for any domain D⊂M, corresponding to the diffeomorphism subgroup $\eta_{t}\in Diff\left(M\right),t\in R,$ generated by flow (71). Taking into account that there holds the following density relationship

(75) $$L_{d/dt}\left(\rho_{t}\left(x_{t}\right)d^{3}x_{t}\right)=0$$

for any t∈R, one easily derives from (74) and (75) that also

(76) $$L_{d/dt}\gamma_{t}\left(m_{t};\rho_{t},\sigma_{t},B_{t}\right)=0$$

for any t∈R. Thus, based on the commutation relationship (73) one can formulate the following important lemma.

**Lemma** **1.** Let vector fields (71) and (72) commute to each other and a density functional $\gamma_{0}:G^{*}\times R\to C^{\infty }\left(M\times R;R\right)$ satisfies for all t∈R the condition

(77) $$L_{d/dt}\gamma_{0}\left(m_{t};\rho_{t},\sigma_{t},B_{t}\right)=0,$$

*then the following expressions*

(78) $$I_{n,k}=\int_{D_{t}}\rho_{t}(L_{u_{t}}^{n}\gamma_{0}{\left(m_{t};\rho_{t},\sigma_{t},B_{t}\right)}^{k}d^{3}x_{t}\;$$

*over the domain $D_{t}=\eta_{t}\left(D\right),$ generated by the corresponding to the flow (71) diffeomorphism subgroup $\eta_{t}\in Diff\left(M\right),t\in R,$ and arbitrary domain D⊂M, are for all integers $n\in Z_{+},$$k\in Z_{,}$ the MHD invariants of the superfluid flow (55).*

**Proof.**  A proof easily follows from the commutation condition (73) and the superfluid density relationship (75). □

As examples let us take, following [33,47], the vector field $u_{t}:=\rho_{t}^{-1}B_{t}\in \Gamma \left(T\left(M\right)\right),$ commuting to the vector field $v_{t}\in \Gamma \left(T\left(M\right)\right),$ and $\gamma_{0}=\;i_{u_{t}}\langle A_{t}\;|dx_{t}\rangle =\langle A_{t}\;|\rho_{t}^{-1}B_{t}\rangle \in C^{\infty }\left(M\times R;R\right),$ where the magnetic vector potential $A_{t}\in C^{\infty }\left(M;R\right),t\in R,$ satisfies the classical Maxwell relationships: the magnetic field $B_{t}=\nabla \times A_{t}$ and the electric field $E_{t}=-\partial A_{t}/\partial t=-v_{t}\times B_{t},$ owing to the net electric field superconductivity (54) condition ${\tilde{E}}_{t}=E_{t}+v_{t}\times B_{t}=0.$ Really, the commutativity condition (73) means that

(79) $$L_{d/dt}\left(\rho_{t}^{-1}B_{t}\right)-\langle \rho_{t}^{-1}B_{t}|\nabla >v_{t}=0,$$

which is satisfied, owing to the second and forth equations of the Euler MHD system (55), as well as to the invariance

(80) $$\begin{array}{c} L_{d/dt}\gamma_{0}=L_{d/dt}i_{u_{t}}\langle A_{t}\;|dx_{t}\rangle =\left[L_{d/dt},i_{u_{t}}\right]\langle A_{t}\;|dx_{t}\rangle +\\ +i_{u_{t}}L_{d/dt}\langle A_{t}\;|dx_{t}\rangle =i_{\left[d/dt,u_{t}\right]}\langle A_{t}\;|dx_{t}\rangle +i_{u_{t}}L_{d/dt}\langle A_{t}\;|dx_{t}\rangle =0, \end{array}$$

which holds owing to the algebraic relationship

(81) $$\;\left[L_{d/dt},i_{u_{t}}\right]=i_{\left[\partial /\partial t+v_{t}v_{t},u_{t}\right]},\;$$

commutativity of vector fields $u_{t}$ and $v_{t}\in \Gamma \left(M\right)$ and the integral relationship

(82) $$\begin{array}{c} \frac{d}{dt}\int_{\partial S_{t}}\langle A_{t}\;|dx_{t}\rangle =\int_{\partial S_{t}}L_{d/dt}\langle A_{t}\;|dx_{t}\rangle =\\ =\int_{\partial S_{t}}\left[\langle L_{d/dt}A_{t}\;|dx_{t}\rangle +\langle A_{t}\;|L_{d/dt}dx_{t}\rangle \right]=\\ =\int_{\partial S_{t}}\left[\langle L_{d/dt}A_{t}\;|dx_{t}\rangle +\langle A_{t}\;|dv_{t}\rangle \right]=\\ =\int_{\partial S_{t}}\left[\langle v_{t}\times B+\langle v_{t}|\nabla \rangle A_{t}\;|dx_{t}\rangle +\langle A_{t}\;|dv_{t}\rangle \right]=\\ =\int_{\partial S_{t}}\left[\langle v_{t}\times \left(\nabla \times A\right)+\langle v_{t}|\nabla \rangle A_{t}\;|dx_{t}\rangle +\langle A_{t}\;|dv_{t}\rangle \right]=\\ =\int_{\partial S_{t}}\left[\langle dA_{t}|v_{t}\rangle +\langle A_{t}\;|dv_{t}\rangle \right]=\int_{\partial S_{t}}\left[d\langle A_{t}\;|v_{t}\rangle \right]=0, \end{array}$$

equivalent to the condition $L_{d/dt}\langle A_{t}\;|dx_{t}\rangle =0$ for all t∈R. The same statement we obtain from the slightly simpler reasoning:

(83) $$\begin{array}{c} \frac{d}{dt}\int_{\partial S_{t}}\langle A_{t}\;|dx_{t}\rangle =\frac{d}{dt}\int_{S_{t}}\langle \nabla \times A_{t}|dS_{t}^{2}\rangle =\\ =\frac{d}{dt}\int_{S_{t}}\langle B_{t}|dS_{t}^{2}\rangle :=-\int_{\partial S_{t}}\langle {\tilde{E}}_{t}|dx_{t}\rangle =0,\; \end{array}$$

following from the net electric field ${\tilde{E}}_{t}=0$ superconductivity condition (54) along the boundary $\partial S_{t},$ where $S_{t}:=\eta_{t}\left(S_{0}\right)\subset M\;$ is the surface, generated by the diffeomorphism subgroup $\eta_{t}\in Diff\left(M\right),t\in R,$ and an arbitrarily chosen surface $S_{0}=S_{t}{|}_{t=0}\subset M.$ The latter is, evidently, equivalent to the equality $L_{d/dt}\langle A_{t}\;|dx_{t}\rangle =0$ modulo the gauge transformation $A_{t}\to$$A_{t}+\nabla \xi_{t},$ where $L_{d/dt}\xi_{t}+\langle A_{t}|v_{t}\rangle$=0 for some function $\xi_{t}\in C^{\infty }\left(M;R\right)$ and all t∈R. Thus, one can formulate [33,47] the following proposition.

**Proposition** **6.** *The functionals*

(84) $$I_{n,k}^{\left(B\right)}=\int_{D_{t}}\rho_{t}{\left(L_{\rho_{t}^{-1}B_{t}}^{n}\langle A|\rho_{t}^{-1}B_{t}\rangle \right)}^{k}d^{3}x_{t}$$

*over the domain $D_{t}=\eta_{t}\left(D\right),$ generated by the corresponding to the flow (71) diffeomorphism subgroup $\eta_{t}\in Diff\left(M\right),t\in R,$ and arbitrary domain D⊂M, are for all integers $n\in Z_{+},k\in Z_{,}$ the MHD invariants of the superfluid flow (55). In particular, the following relationships $\{H,I_{n,k}^{\left(B\right)}\}=0$ hold for all $n\in Z_{+}.$*

**Remark** **1.** *It is natural here to mention [33,35] that the specific entropy functional $\gamma_{0}=\sigma_{t}:M$→$C^{\infty }\left(M\times R;R\right)\;\;$ satisfies the sufficient condition $L_{d/dt}\sigma_{t}=0,t\in R,$ a priori generates for the superfluid flow (55) the infinite hierarchy*

(85) $$I_{n,k}^{\left(\sigma \right)}=\int_{D_{t}}\rho_{t}{\left(L_{\rho_{t}^{-1}B_{t}}^{n}\sigma_{t}\left(x_{t}\right)\right)}^{k}d^{3}x_{t},\;$$

*$n\in Z_{+},$k∈Z, of the MHD invariants over the domain $D_{t}=\eta_{t}\left(D\right),$ generated by the corresponding to the flow (71) diffeomorphism subgroup $\eta_{t}\in Diff\left(M\right),t\in R,$ and arbitrary domain D⊂M.*

To construct other MHD invariants, depending on the superfluid velocity $v_{t}\in \Gamma \left(T\left(M\right)\right),$t∈R, let us consider, following [47], two differential one-forms $\langle \alpha_{t}|dx_{t}\rangle ,\langle \beta_{t}|dx_{t}\rangle \in \Lambda^{1}\left(M\right),$$x_{t}:=\eta_{t}\left(X\right),$X∈M, satisfying for all t∈R the following identity:

(86) $$L_{d/dt}\langle \alpha_{t}|dx_{t}\rangle =dh_{t}\;\;+L_{u_{t}}\langle \beta_{t}|dx_{t}\rangle ,\;$$

for some function $h_{t}\in \Lambda^{0}\left(M\right),$ where the vector field

(87) $$dx_{t}/d\tau =\;u_{t}\left(x_{t}\right)$$

is uniform with respect to the evolution parameter τ∈R and satisfies the following constraints:

(88) $$\left[L_{d/dt},L_{u_{t}}\right]=0,\;\;\;\langle \nabla |\rho_{t}u_{t}\;\rangle =0\;\;\;\;$$

and $u_{t}\|\partial M$ at almost all points $x_{t}\in \partial M$ for all evolution parameters t,τ∈R. Then one can formulate the following general proposition.

**Proposition** **7.** *The following integral expressions*

(89) $$\begin{array}{ccc} I_{0}^{\left(\alpha ,\beta \right)} & = & \int_{M}\rho_{t}\langle \alpha_{t}|u_{t}\rangle d^{3}x_{t},I_{1}^{\left(\alpha ,\beta \right)}=\int_{M}\rho_{t}\left[\langle \alpha_{t}|v_{t}\rangle +h_{t}\right]d^{3}x_{t},\\ I_{2}^{\left(\alpha ,\beta \right)} & = & \int_{M}\rho_{t}\langle L_{d/dt}\alpha_{t}|u_{t}\rangle d^{3}x_{t} \end{array}$$

*over the whole domain $M\subset R^{3}$ are for all integers $n\in Z_{+},$k∈Z, the global MHD invariants.*

**Proof.**  Consider, for example, a proof that $I_{0}^{\left(\alpha ,\beta \right)}:G\to R$ is an invariant: taking into account that $L_{d/dt}\left(\rho_{t}d^{3}x_{t}\right)=0,$ one obtains the expression:

(90) $$\begin{array}{c} dI_{0}^{\left(\alpha ,\beta \right)}/dt=\int_{M}\rho_{t}L_{d/dt}\langle \alpha_{t}|u_{t}\rangle d^{3}x_{t}\;=\\ =\int_{M}\rho_{t}i_{u_{t}}\left(dh_{t}+L_{u_{t}}\langle \beta_{t}|dx_{t}\rangle \right)d^{3}x_{t}=\\ =\int_{M}\rho_{t}\left(i_{u_{t}}dh_{t}+i_{u_{t}}\left(i_{u_{t}}d+di_{u_{t}}\right)\langle \beta_{t}|dx_{t}\rangle \right)d^{3}x_{t}=\\ =\int_{M}\rho_{t}i_{u_{t}}d\left(h_{t}+\langle \beta_{t}|u_{t}\rangle \right)d^{3}x_{t}=\\ =\int_{M}\langle \nabla |{\tilde{h}}_{t}\rho_{t}u_{t}\rangle d^{3}x_{t}=\int_{\partial M}\langle {\tilde{h}}_{t}\rho_{t}u_{t}|dS_{t}^{2}\rangle =0\; \end{array}$$

for all t∈R, where we put, by definition, ${\tilde{h}}_{t}:=\;\left(h_{t}+\langle \beta_{t}|u_{t}\rangle \right),\;$ denoted $dS_{t}^{2}$ the surface measure on the boundary ∂M, used the Cartan representation $L_{u_{t}}=i_{u_{t}}d+di_{u_{t}}\;$ and the natural boundary tangency condition $\rho_{t}{u_{t}}_{\left|\right|}\partial M,$ thus proving the proposition. Exactly similar calculations ensue for the next two invariant $I_{k}^{\left(\alpha ,\beta \right)}:G\to R,k=\bar{1,2},$ on which we will not stop here. □

As a simple example, one can put $\alpha_{t}^{\left(0\right)}:=♭\left(v_{t}\right)≃v_{t},\beta_{t}:=B_{t},$ the vector field $u_{t}=\rho_{t}^{-1}B_{t}:M\to T\left(M\right),t\in R,$ and show by easy calculations, using the variational equality (56) that

(91) $$L_{d/dt}\langle v_{t}|dx_{t}\rangle =d(|v_{t}{|}^{2}/2\;-h_{t}-|B_{t}{|}^{2}/\rho_{t})+L_{u_{t}}\langle B_{t}|dx_{t}\rangle +T_{t}d\sigma_{t},\;$$

where, we have denoted the specific enthalpy [41,42,43] function $h_{t}:=e_{t}+p_{t}\rho_{t}^{-1}.$ As a consequence of equality (91), under the spatial temperature constancy $\nabla T_{t}=0$ condition for all t∈R, one obtains the following MHD superfluid invariant:

(92) $$I_{0}^{\left(v,B\right)}:=\int_{M}\;\langle v_{t}|B_{t}\rangle d^{3}x_{t}=\;\int_{M}\;\langle m_{t}|\rho_{t}^{-1}B_{t}\rangle ,$$

where $m_{t}\;≃$$\langle m_{t}\left(x_{t}\right)|dx_{t}\rangle ⊗d^{3}x_{t}\in diff^{*}\left(M\right)$ and $\rho_{t}^{-1}B_{t}≃\langle \rho_{t}^{-1}\left(x\right)B_{t}|\partial /\partial x\rangle$∈T(M), coinciding with the MHD invariant, presented before in [33,47]. If the above temperature condition does not hold, the equality (91) reduces to the differential relationship

(93) $$\partial \langle v_{t}|B_{t}\rangle /\partial t+\langle \nabla |[v_{t}\langle v_{t}|B_{t}\rangle +B_{t}(h_{t}-|v_{t}{|}^{2}/2]\rangle +\rho_{t}T_{t}\langle \rho_{t}^{-1}B_{t}|\nabla \sigma_{t}\rangle ,$$

satisfied for all $x_{t}\in M$ and t∈R.

**Remark** **2.** *It is worth to remark here that the following baroclinic relationship*

(94) $$\nabla \rho_{t}^{-1}\times \nabla p_{t}=-\nabla T_{t}\times \nabla \sigma_{t}$$

*holds for all $x_{t}\in M$ and t∈R.*

Similarly we also easily obtain the following invariant

(95) $$I_{1}^{\left(v,B\right)}=\;\int_{M}\;\rho_{t}\left[\right|m_{t}{|}^{2}/\left(2\rho_{t}^{2}\right)+w_{t}^{\left(0\right)}\left(\rho_{t},\sigma_{t}\right)+|B_{t}{|}^{2}/\left(2\rho_{t}\right)]d^{3}x_{t}=H,\;$$

coinciding exactly with the Hamiltonian function for the flow (55) on the phase space $G^{*}.$ The third invariant is, eventually, closely related to the vorticity vector $\xi_{t}:=\nabla \times v_{t}:M\to E^{3},t\in R,$ and needs a more detail analysis.

It is instructive now to analyze the existence of integral invariants for the pure hydrodynamic case when the magnetic field $B_{t}=0,$t∈R, following the approach, devised before in [47]. In particular, owing to the relationship (94), there holds the following integral expression for the vorticity $\xi_{t}:=\nabla \;\times v_{t},t\in \;R:$

(96) $$L_{d/dt}\xi_{t}-\langle \xi_{t}|\nabla \rangle v_{t}=\nabla T_{t}\times \nabla \sigma_{t}\;$$

and define the vector field

(97) $$u_{t}:=\rho_{t}^{-1}\xi_{t}\exp f_{t}\left(x_{t}\right)$$

for some scalar smooth mapping $f_{t}:M\to R,$ which we will choose from the assumed commutation condition

(98) $$[L_{d/dt},L_{u_{t}}]=0.$$

The latter gives rise to the equality $\;\;\;\xi_{t}L_{d/dt}f_{t}\left(x_{t}\right)=-\nabla T_{t}\times \nabla \sigma_{t}$ at any $x_{t}:=\eta_{t}\left(X\right)$∈M,X∈M, or

(99) $${\dot{f}}_{t}\;\left(\nabla \times v_{t}\right)+\nabla T_{t}\times \nabla \sigma_{t}=0,$$

where we took into account that $L_{d/dt}f_{t}\left(x_{t}\right)=df_{t}\left(x_{t}\right)/dt:={\dot{f}}_{t}\left(x_{t}\right),\;$$x_{t}$∈M, with respect the temporal parameter t∈R. From (99) one obtains that the mapping $f_{t}:M\to R\;$ should satisfy the following constraints:

(100) $$\nabla {\dot{f}}_{t}=k_{t}v_{t},\;\;\;{\dot{f}}_{t}v_{t}=\rho_{t}^{-1}\nabla p\left(t\right)+\nabla \omega_{t}$$

for some scalar smooth functions $k_{t}$ and $\omega_{t}$:M→R,t∈R. It is easy to check that the system (100) is compatible, i.e., the quasi-stationary thermodynamic relationship $p_{t}^{\left(0\right)}=\rho_{t}^{2}\partial w_{0}\left(\rho_{t},\sigma_{t}\right)/\partial \rho_{t}$ jointly with Euler Equation (10) make it possible to determine these unknown scalar smooth functions $k_{t}$ and $\omega_{t}$:M→R for all t∈R.

Consider now, following [47], a slightly modified expression (91) at the magnetic field $B_{t}=0:$

(101) $$L_{d/dt}\langle v_{t}\exp f_{t}|dx_{t}\rangle =\exp f_{t}d(\omega_{t}+|v_{t}{|}^{2}/2)\;$$

and calculate the related integral expression:

(102) $$\begin{array}{c} \frac{d}{dt}\int_{M}\rho_{t}\left(i_{u_{t}}\langle v_{t}|dx_{t}\rangle \right)d^{3}x_{t}\;=\int_{M}\rho_{t}L_{d/dt}\left(i_{u_{t}}\langle v_{t}|dx_{t}\rangle \right)d^{3}x_{t}=\\ =\int_{M}\rho_{t}\left(i_{u_{t}}L_{d/dt}\langle v_{t}|dx_{t}\rangle \right)d^{3}x_{t}=\int_{M}\rho_{t}\left(i_{u_{t}}d\tilde{h}\right)d^{3}x_{t}=\\ =\int_{M}\left(i_{\rho_{t}u_{t}}d\tilde{h}\right)d^{3}x_{t}=\int_{M}\langle \nabla {\tilde{h}}_{t}|\rho_{t}u_{t}\rangle d^{3}x_{t}=\int_{M}\langle \nabla {\tilde{h}}_{t}|\xi_{t}\exp f_{t}\left(x_{t}\right)\rangle d^{3}x_{t}, \end{array}$$

where we put, by definition, the function ${\tilde{h}}_{t}:=\omega_{t}+{\left|v_{t}\right|}^{2}/2.$

If now to put that the mapping $f_{t}:M\to$R satisfies for all t∈R the constraint $\langle \nabla f_{t}|\xi_{t}\rangle =0,$ the integral expression (102) reduces to

(103) $$\begin{array}{c} \frac{d}{dt}\int_{M}\rho_{t}\;\left(i_{u_{t}}\langle v_{t}|dx_{t}\rangle \right)d^{3}x_{t}=\int_{M}\langle \nabla |\left(\exp f_{t}\left(x_{t}\right){\tilde{h}}_{t}\xi_{t}\right)\rangle d^{3}x_{t}=\\ =\int_{\partial M}\langle \exp f_{t}\left(x_{t}\right){\tilde{h}}_{t}\xi_{t}|d^{2}S_{t}\rangle =0, \end{array}$$

where there is assumed the vorticity vector tangency $\xi_{t}\left|\right|\partial M$ constraint. Thus, under conditions assumed above, the following vortex functional

(104) $$I=\int_{M}\langle v_{t}|\nabla \times v_{t}\rangle d^{3}x_{t}$$

persists to be conserved for all t∈R.

If the function $f_{t}:M\to$R, being defined by relationships (100), satisfies for all t∈R the scalar constraint $\langle \nabla f_{t}|\xi_{t}\rangle =0,$ one easily derives the following differential relationship:

(105) $$\begin{array}{ccc} L_{d/dt}\langle \nabla f_{t}|\xi_{t}\rangle & = & k_{t}\langle v_{t}|\xi_{t}\rangle +\langle \nabla |f_{t}\nabla T_{t}\times \nabla \sigma_{t}\rangle =\\ & = & <\nabla {\dot{f}}_{t}|\xi_{t}\rangle +\langle \nabla |f_{t}\nabla T_{t}\times \nabla \sigma_{t}\rangle =0, \end{array}$$

or, equivalently, in the integral form

(106) $$\begin{array}{c} \frac{d}{dt}\int_{D_{t}}\langle \nabla f_{t}|\xi_{t}\rangle \rho_{t}d^{3}x_{t}=\int_{D_{t}}L_{d/dt}\langle \nabla f_{t}|\xi_{t}\rangle \rho_{t}d^{3}x_{t}=\\ =\int_{D_{t}}\left[\langle \nabla {\dot{f}}_{t}|\xi_{t}\rangle +\langle \nabla |f_{t}\nabla T_{t}\times \nabla \sigma_{t}\rangle \right]\rho_{t}d^{3}x_{t}=\\ =\int_{D_{t}}\left[\langle \nabla {\dot{f}}_{t}|\xi_{t}\rangle -\langle \nabla f_{t}|\nabla \times \rho_{t}^{-1}\nabla p_{t}^{\left(0\right)}\rangle \right]\rho_{t}d^{3}x_{t}\\ =\int_{D_{t}}\left[\langle \nabla {\dot{f}}_{t}|\xi_{t}\rangle \rho_{t}-\rho_{t}\langle \nabla \rho_{t}^{-1}|\nabla \times p_{t}^{\left(0\right)}\nabla f_{t}\rangle \right]d^{3}x_{t}=\\ =\int_{D_{t}}\left[\langle \nabla {\dot{f}}_{t}|\xi_{t}\rangle \rho_{t}+\langle \nabla \ln\rho_{t}|\nabla \times p_{t}^{\left(0\right)}\nabla f_{t}\rangle \right]d^{3}x_{t}=\\ =\int_{D_{t}}\langle \nabla {\dot{f}}_{t}|\xi_{t}\rangle \rho_{t}d^{3}x_{t}, \end{array}$$

where we took into account that for the isentropic fluid flow under regard there holds the tangency $\;\nabla \rho_{t}\left|\right|\partial D_{t}$ condition for all t∈R. If the right hand side of (106) proves to be zero, i.e., $\langle \nabla \;{\dot{f}}_{t}|\xi {\;}_{t}\rangle \;=0,t\in R,$ this will mean that the constraint $\langle \nabla f_{t}|\xi_{t}\rangle =0$ for all t∈R, if ${\left.\langle \nabla f_{t}|\xi_{t}\rangle \right|}_{t=0}=0$ at t=0, thus producing the vortex conservation functional (104).

## 7. The Isentropic Flows on Phase Spaces with Gauge Symmetry

In this section, we are interested in description of isentropic charged liquid flows on phase spaces with gauge symmetry, imposed by an external electromagnetic field. Unlike Section, where the external magnetic field was completely frozen into the charged superfluid and completely governed by its dynamics, the case under present regard strongly differs from the latter and should take into account two independent yet interacting dynamic systems. As the phase space under regard is endowed with gauge type electromagnetic field symmetry, the common dynamics of the coupled fluid and electromagnetic field should be properly considered on the related principal fiber bundle $P(Q_{em},F)$ over the reduced fluid base space $Q_{em}≃$$(\Lambda^{2}\left(M\right)\times d\Lambda^{1}\left(M\right))\times$$diff^{*}\left(M\right)$$\times (\Lambda^{3}\left(M\right)\times \Lambda^{0}\left(M\right))$ with the abelian structure group $F≃d\Lambda^{0}\left(M\right),$ acting on the fiber bundle *P* from the right via the gauge type transformation. We assume that locally the principal fiber bundle $P\left(Q_{em},F\right){|}_{loc}≃T^{*}\left(G\right)\times \left(*\Lambda^{1}\left(M\right)\times \Lambda^{1}\left(M\right)\right),$ where the group $G=Diff\left(M\right)⋉(\Lambda^{0}\left(M\right)\times \Lambda^{3}\left(M\right)),$ the space $\Lambda^{1}\left(M\right)$ models the magnetic vector potential on M, its factor space $\Lambda^{1}\left(M\right)/F≃$$d\Lambda^{1}\left(M\right)\subset \Lambda^{2}\left(M\right)$ models the ambient magnetic field on M, the product $T^{*}\left(G\right)\times \left(*\Lambda^{1}\left(M\right)\times \Lambda^{1}\left(M\right)\right)$ of cotangent spaces models the moving charged liquid under the ambient electromagnetic field and determines the Hamiltonian function

(107) $$H_{em}=\int_{M}d^{3}{x(|\mu -\theta \rho A|}^{2}/\left(2\rho \right)+\rho w^{\left(0\right)}\left(\rho ,\sigma \right)+{\left(|E\right|}^{2}{+|\nabla \times A|}^{2})/2),$$

where $E:=-Y,\left(Y,A\right)\in *\Lambda^{1}\left(M\right)\times \Lambda^{1}\left(M\right)),\mu =\rho v,$$\left(\mu ;\rho ,\sigma \right)\in T^{*}\left(G\right),$$w^{\left(0\right)}:T^{*}\left(G\right)$$\to C^{\infty }\left(M;R\right)$ denotes the internal potential energy function and $\theta \in R_{+}$ denotes the corresponding charge/mass ratio of the fluid under regard. The resulting evolution equations of the liquid motion with respect to the temporal parameter t∈R look in noncanonical variables as follows:

(108) $$\begin{array}{ccc} \partial v/\partial t+\langle v|\nabla \rangle v & = & \theta \left(E+v\times B\right)-\rho^{-1}\nabla p^{\left(0\right)},\\ \partial \rho /\partial t+\langle \nabla |\rho v\rangle & = & 0,\;\;\;\;\partial \sigma /\partial t+\langle \nabla |v\rangle \sigma =0,\langle \nabla |E\rangle =\theta \rho ,\\ \;\;\;\partial E/\partial t & = & \nabla \times B-\theta \rho v,\;\;B=\nabla \times A, \end{array}$$

where, in general, the pressure $p^{\left(0\right)}:=\rho^{2}(\partial w^{\left(0\right)}\left(\rho ,\sigma \right)/\partial \rho$$+\sigma /\rho \partial w^{\left(0\right)}\left(\rho ,\sigma \right)/\partial \sigma )$ and phase space points $\left(E,B;\rho v;\rho ,\sigma \right)\in Q_{em}\;$ belong here to the base manifold of the fiber bundle $P(Q_{em},F).$

To proceed further in more detail, we begin by reviewing some backgrounds of the reduction theory subject to Hamiltonian systems with symmetry on principle fiber bundles. Some of the material is partly available in [7,19,30,48], so here it will be only sketched in notations suitable for us.

Consider a principal fiber bundle $P(Q_{em},F)$ over the base space $Q_{em}$ with the projection $\pi :P\to Q_{em}\;$ and the abelian structure group F≃$\Lambda^{0}\left(M\right),\;$ acting from the right on *P* by means of a smooth mapping χ:F×P→P. Taking into account that $\Lambda^{1}\left(M\right)≃\Lambda^{2}\left(M\right)≃$$d\Lambda^{1}\left(M\right)⊕\Lambda^{2}\left(M\right)/d\Lambda^{1}\left(M\right),$$\;\Lambda^{2}\left(M\right)/d\Lambda^{1}\left(M\right)≃d\Lambda^{0}\left(M\right)≃$ mathcalF, owing to the classical [4,31,49] Helmgoltz representation, for each h∈F a group diffeomorphism $\chi_{h}:P\to P$ generates for any fixed u∈P the induced mapping $\hat{u}:F\to P,$ where

(109) $$\;\hat{u}\left(h\right):=\chi_{h}\left(u\right)\;$$

for all h∈F, being equal to the usual gauge transformed expression

(110) $$\hat{u}\left(h\right)=\left(\mu +\theta \rho \left(x\right)d\ln h\right];\rho \left(x\right)d^{3}x,\sigma ;\left(*\langle Y|dx\rangle ,\langle A|dx\rangle +d\ln h\right)),$$

where we made used of the local coordinate representation of *P* in coordinates $u:=(\mu ;\rho \left(x\right)d^{3}x,\sigma ;\left(*\langle Y|dx\rangle ,\langle A|dx\rangle \right))$$\in P,\;\pi (R_{h}u)=$$\pi \left(u\right):=(\mu ;\rho \left(x\right)d^{3}x,\sigma ;$$(*\langle {\left(\nabla \times \right)}^{-1}Y|dx\rangle ,\langle B|dx\rangle )),$x∈M, for any h∈F,π(A):=B=∇×A and suitable vector $\;Y\in C^{\infty }\left(M;E^{3}\right).\;$ We here also assume that the gauge type transformation $\chi_{h}:P\to P,$h∈F, is equivariant [5,7,31,44] subject to the canonical product Poisson bracket {·,·}:=${\left\{\cdot ,\cdot \right\}}_{T^{*}\left(G\right)}⊗$${\left\{\cdot ,\cdot \right\}}_{T^{*}\left(\Lambda^{1}\left(M\right)\right)}$ on P, that is for any smooth functionals $g_{1},g_{2}:P\to R$ the invariance relationship

(111) $$\left\{g_{1}\circ \chi_{h},g_{2}\circ \chi_{h}\right\}=\left\{g_{1},g_{2}\right\}\circ \chi_{h}$$

holds for all h∈F.

Let e∈F be the unit element of the structure group *F* and denote by $d\Lambda^{0}\left(M\right)\;$≃F the corresponding Lie algebra (abelian) of the structure group *F* as the tangent space $T_{e}\left(F\right)≃\;$ mathcalF at e∈F. The tangent mapping to (109) acts as ${\hat{u}}_{*}\left(e\right):$ mathcalF $\to T_{u}\left(P\right)$ for u∈P and is equal in local coordinates to the tangent vector expression

(112) $${\hat{u}}_{*}\left(e\right)\left(df\right)=\left(\theta df⊗\rho \left(x\right)d^{3}x;0,0;\left(0,\langle \nabla f|dx\rangle \right)\right)\;$$

at u∈P, where df∈ mathcalF $≃d\Lambda^{0}\left(M\right)$ and x∈M, where we took into account the corresponding action of the abelian group *F* on the group The mapping (110) makes it possible to define on the principal fiber bundle $P(T^{*}\left(G_{em}\right),F)$ a connection Γ(A) by means of constructing [10,11,50] a morphism A$:(T\left(P\right),\chi_{h,*})\to$(mathcalF$,Ad_{h^{-1}}),h\in F,$ such that for each u∈P the corresponding mapping $A\left(u\right):T_{u}\left(P\right)\;\to$ mathcalF is a left inverse one to the mapping

(113) $${\hat{u}}_{*}\left(e\right):F\to T_{u}\left(P\right),$$

that is

(114) $$A\left(u\right){\hat{u}}_{*}\left(e\right)=Id,\;$$

where, by definition, for some vector-functions Φ and $Z\in C^{\infty }\left(P;E^{3}\right)$

(115) A(u)=〈dΦ|δμ〉+〈∗(dx−∇×dZ−θρdΦ)|〈δA|dx〉〉

at u∈P,x∈M. Really, by definition, for any fundamental vertical vector field

(116) $$d\tilde{f}={\hat{u}}_{*}\left(e\right)\left(df\right)=\left(\theta df⊗\rho \left(x\right)d^{3}x;0,0;\left(0,\langle \nabla f|dx\rangle \right)\right),$$

generated by an element ∇lnh≃df∈ mathcalF, there should be $\langle A\left(u\right)|d\tilde{f}\rangle =df:$

(117) $$\begin{array}{c} \langle A\left(u\right)|d\tilde{f}\rangle =\langle *\left(\theta df\right)⊗\rho \left(x\right)d^{3}x|d\Phi \rangle +\\ +\langle \left(dx-\theta \rho d\Phi \right)|\nabla f\rangle d^{3}x≃\;\langle dx|\nabla f\rangle =df, \end{array}$$

being completely satisfied. The needed invariance $R_{h*}A\left(u\right)=Ad_{h^{-1}}A\left(u\right)$ is also satisfied automatically for any h∈F.

The induced by mapping (113) Lie algebra action $\;{\hat{u}}_{*}\left(e\right)\;:F\times P\to T_{u}\left(P\right)\;$ naturally generates [9,31,36,38,51] the *momentum mapping*$l:T_{u}^{*}\left(P\right)\to$ mathcalF${}^{*}$ at u∈P for any f∈F that for the vertical vector field $\tilde{df}=d\langle A|dx\rangle /d\tau$=〈∇f|dx〉∈ mathcal F

(118) $$\begin{array}{c} \langle l\left(\alpha^{\left(1\right)}\left(u\right)\right)|\tilde{df}\rangle =\langle {\hat{u}}^{*}\left(e\right)\alpha^{\left(1\right)}\left(u\right)|\langle \nabla f|dx\rangle \rangle =\\ =\langle \alpha^{\left(1\right)}\left(u\right)|{\hat{u}}^{*}\left(e\right)\langle \nabla f|dx\rangle \rangle =\;\langle J\left(\mu ;\rho \right)+Y|\nabla f\rangle d^{3}x, \end{array}$$

where $\;\alpha^{\left(1\right)}\left(u\right):=\langle *\langle Y|dx\rangle |\langle \delta A|dx\rangle \rangle +\langle \alpha \left(\mu ;\rho ,\sigma \right)|{\left(\delta \mu ;\delta \rho ,\delta \sigma \right)}^{⊺}\rangle \in T_{u}^{*}\left(P\right)$ for some element α(μ;ρ,σ)∈$T^{*}\left(T^{*}\left(G_{em}\right)\right)$ is the corresponding Liouville form on the cotangent bundle $T^{*}\left(G_{em}\right),$ the following determining equalities hold:

(119) $$\begin{array}{ccc} H_{f}\left(\mu ;\rho \right)d^{3}x\; & : & =\langle *\langle J(\mu ;\rho )|dx\rangle |\langle \nabla f|dx\rangle \rangle ,\\ {\left(\theta \rho \left(x\right)\nabla f;0,0\right)}^{⊺} & : & ={\left\{H_{f}\left(\mu ;\rho \right),{\left(\mu ;\rho ,\sigma \right)}^{⊺}\right\}}_{T^{*}\left(G\right)} \end{array}$$

with respect to the canonical Lie-Poisson bracket (29) on the cotangent bundle $T^{*}\left(G\right)$ to the group manifold $G=Diff\left(M\right)⋉(\Lambda^{0}\left(M\right)\times \Lambda^{3}\left(M\right)).$

Fix now the momentum mapping value

$$l\left(\alpha^{\left(1\right)}\left(u\right)\right)=*\langle J\left(\mu ;\rho \right)+Y|dx\rangle :=*\langle \xi |dx\rangle \in F^{*},$$

equivalent to the condition $-\langle \nabla |\xi \rangle =\bar{\xi }\in C^{\infty }\left(M;R\right),$ construct the submanifold $P_{\xi }:=\{u\in P:l\left(\alpha^{\left(1\right)}\left(u\right)\right)$=ξ∈ mathcalF${}^{*}\}$ and consider the reduced phase space ${\bar{P}}_{\xi }:=P_{\xi }/F_{\xi },$ where $F_{\xi }:=\{h\in F:Ad_{h}^{*}$∗〈ξ|dx〉=∗〈ξ|dx〉} is the stationary subgroup of the element ξ∈ mathcalF${}^{*}.$ Taking into account that in our case $F_{\xi }=F\;$ for any element ξ∈ mathcalF${}^{*},$ one can formulate the following theorem, characterizing [38,50] the related gauge symmetry symplectic structure reduction on the reduced manifold $\bar{P}{}_{\xi },\xi \in$ mathcalF${}^{*}.$

**Theorem** **1.** Given a principal fiber F-bundle with a connection Γ(A) on the principal fiber bundle $P(Q_{em};F)$ and an F-invariant element $\;\xi \in \;F^{*},$ then every such connection Γ(A) defines a symplectomorphism $\nu_{\xi }:\bar{P}{}_{\xi }\to Q_{em}$ between the reduced phase space ${\bar{P}}_{\xi }$ and the base manifold $Q_{em}.$ Moreover, the following equality

(120) $$\left(d\;\beta_{Q_{em}}^{\left(1\right)}+\;\Omega_{\xi }^{\left(2\right)}{|}_{Q_{em}}\right)={\left.\nu_{\xi }^{*}\;\alpha^{\left(1\right)}\right|}_{\bar{P}{}_{\xi }}$$

*holds for the canonical Liouville forms $\beta^{\left(1\right)}\in \Lambda^{1}\left(Q_{em}\right)$ and $\alpha^{\left(1\right)}\in \Lambda^{1}\left(P\right),$ where $\Omega_{\xi }^{\left(2\right)}:=\langle \xi |\Omega^{\left(2\right)}\rangle$ is the ξ-component of the corresponding curvature form $\Omega^{\left(2\right)}\in \Lambda^{\left(2\right)}\left(Q_{em}\right))⊗F.$*

The statements above make it possible to construct a true symplectic structure on the cotangent bundle $T^{*}\left(G_{em}\right).$ Namely, making use of the connection form (115) and symplectic structure expression (120), one derives the resulting reduced symplectic structure on the base manifold $Q_{em}:$

(121) $$\begin{array}{c} \omega_{\xi }^{\left(2\right)}\left(\pi \left(u_{\xi }\right)\right)=\langle \delta \alpha |\wedge {\left(\delta \mu ;\delta \rho ,\delta \sigma \right)}^{⊺}\rangle {|}_{\mu \to \mu -\theta \rho A}+\\ +\langle \nabla |\xi \rangle \langle \delta Z\wedge \delta B\rangle +\langle {\left(\nabla \times \right)}^{-1}\delta Y|\wedge \delta B\rangle +\\ +\langle {\left(\nabla \times \right)}^{-1}\left[\theta \langle \nabla |\xi \rho \rangle \delta \Phi +\theta \langle \nabla |\xi \delta \rho \rangle \Phi \right]|\wedge \delta B\rangle =\omega^{\left(2\right)}{\left(\mu ;\rho ,\sigma \right)|}_{\mu \to \mu -\theta \rho A}+\\ +\langle \langle \nabla |\xi \rangle \delta Z+{\left(\nabla \times \right)}^{-1}\left[\delta Y+\theta \langle \nabla |\xi \rho \rangle \delta \Phi +\theta \langle \nabla |\xi \delta \rho \rangle \Phi \right]|\wedge \delta B\rangle \end{array}$$

at $\pi \left(u_{\xi }\right)=\left(Y,B\right)\times \left(\mu ;\rho ,\sigma \right)\in Q_{em}$ for the fixed element $*\langle \xi |dx\rangle \in F^{*},$ where the expression $\omega^{\left(2\right)}\left(\mu ;\rho ,\sigma \right):=\langle \delta \alpha |\wedge {\left(\delta \mu ;\delta \rho ,\delta \sigma \right)}^{⊺}\rangle$ is generated by the canonical Lie-Poisson bracket (29). The reduced Poisson structure on the base manifold $Q_{em},$ corresponding to the symplectic structure (121) and calculated at the vanishing vectors Z=0,Φ=0, can be easily written down as

(122) $$\begin{array}{ccc} & & \begin{array}{c} \left\{g_{1},g_{2}\right\}\left(\mu ;\rho ,\sigma ;E,B\right)=\int_{M}d^{3}x\left[\langle \mu |\langle \frac{\delta g_{2}}{\delta \mu }|\nabla \rangle \frac{\delta g_{1}}{\delta \mu }-\langle \frac{\delta g_{1}}{\delta \mu }|\nabla \rangle \frac{\delta g_{2}}{\delta \mu }\rangle \right]+\\ +\int_{M}d^{3}x\rho \left[\langle \frac{\delta g_{2}}{\delta \mu }|\nabla \frac{\delta g_{1}}{\delta \rho }\rangle -\langle \frac{\delta g_{1}}{\delta \mu }|\nabla \frac{\delta g_{2}}{\delta \rho }\rangle \right]+ \end{array}\\ & & \begin{array}{c} +\int_{M}d^{3}x\sigma \left[\langle \frac{\delta g_{2}}{\delta \mu }|\nabla \frac{\delta g_{1}}{\delta \sigma }\rangle -\langle \frac{\delta g_{1}}{\delta \mu }|\nabla \frac{\delta g_{2}}{\delta \sigma }\rangle \right]+\\ +\int_{M}d^{3}x\left[\langle \frac{\delta g_{1}}{\delta E}|\nabla \times \frac{\delta g_{2}}{\delta B}\rangle -\langle \frac{\delta g_{2}}{\delta E}|\nabla \times \frac{\delta g_{1}}{\delta B}\rangle \right]+\\ +\int_{M}d^{3}x\left[\langle \frac{\delta g_{1}}{\delta \mu }|\frac{\delta g_{2}}{\delta E}\rangle -\langle \frac{\delta g_{2}}{\delta \mu }|\frac{\delta g_{1}}{\delta E}\rangle +\theta \rho \langle B|\frac{\delta g_{1}}{\delta \mu }\times \frac{\delta g_{2}}{\delta \mu }\rangle \right] \end{array} \end{array}$$

for any smooth functionals $g_{1},g_{2}:P\to R,$ where $\left(\mu ;\rho ,\sigma ;E,B\right)\in Q_{em}.$ The related with the Hamiltonian function (107) subject to the Poisson bracket (122) evolution equations

(123) $$\frac{\partial }{\partial t}{\left(\mu ;\rho ,\sigma ;E,B\right)}^{⊺}=\left\{H_{em},{\left(\mu ;\rho ,\sigma ;E,B\right)}^{⊺}\right\}$$

coincide exactly with those (108), constructed directly from the classical mechanics and electromagnetic laws. We need to remark here that the Poisson bracket structure, related with the obtained above reduced symplectic structure (121) on the base manifold $Q_{em},$ generalizes the Poisson bracket [7,19], and can be eventually used for analyzing nonregular charged fluid dynamics with singularities, including vortices and boundary topological peculiarities. It is easily also generalized to describing a multicomponent charged liquid dynamics.

## 8. Conclusions

In our review, we presented a detailed enough differential geometric description of the isentropic fluid motion phase space and featuring it in the diffeomorphism group structure, modelling the related dynamics, as well as its compatibility with the quasi-stationary thermodynamical constraints. There was analyzed the adiabatic liquid dynamics, within which, following the general approach, the nature of the related Poissonian structure on the fluid motion phase space, as a semidirect Banach groups product, and a natural reduction of the canonical symplectic structure on its cotangent space to the classical Lie-Poisson bracket on the adjoint space to the corresponding semidirect Lie algebras product is explained in detail. We also presented a modification of the Hamiltonian analysis in the case of isothermal liquid dynamics. Some material was devoted to studying the differential-geometric structure of the adiabatic magneto-hydrodynamic superfluid phase space and its related motion within the Hamiltonian analysis and invariant theory. In particular, we constructed an infinite hierarchy of different kinds of integral magneto-hydrodynamic invariants, generalizing those previously constructed in [33,35], and analyzed their differential-geometric origins. We also investigated charged liquid dynamics on the phase space invariant with respect to an abelian gauge group transformation.
